# What Determines the Assembly of Transcriptional Network Motifs in *Escherichia coli*?

**DOI:** 10.1371/journal.pone.0003657

**Published:** 2008-11-06

**Authors:** Francisco M. Camas, Juan F. Poyatos

**Affiliations:** Logic of Genomic Systems Laboratory, Spanish National Biotechnology Centre, Consejo Superior de Investigaciones Científicas (CSIC), Madrid, Spain; Center for Genomic Regulation, Spain

## Abstract

Transcriptional networks are constituted by a collection of building blocks known as network motifs. Why do motifs appear? An adaptive model of motif emergence was recently questioned in favor of neutralist scenarios. Here, we provide a new picture of motif assembly in *Escherichia coli* which partially clarifies these contrasting explanations. This is based on characterizing the linkage between motifs and sensing or response specificity of their constituent transcriptional factors (TFs). We find that sensing specificity influences the distribution of autoregulation, while the tendency of a TF to establish feed-forward loops (FFLs) depends on response specificity, i.e., regulon size. Analysis of the latter pattern reveals that coregulation between large regulon-size TFs is common under a network neutral model, leading to the assembly of a great number of FFLs and bifans. In addition, neutral exclusive regulation also leads to a collection of single input modules -the fourth basic motif. On the whole, and even under the conservative neutralist scenario considered, a substantial group of regulatory structures revealed adaptive. These structures visibly function as fully-fledged working units.

## Introduction

The collection of transcriptional interactions in a cell constitutes a network able to sense diverse biochemical signals and execute, in response, a range of cellular programs. Recent analyses of this network revealed a series of strategies of cellular control at the system-level, which have later shown to be applicable to other classes of biological networks [1].

More specifically, the successive analysis of *Escherichia coli*'s transcriptional network, where interactions involve a pair of operons encoding the transcription factor (TF) and regulated genes [text S1 section 1], respectively [2], identified the presence of a number of recurrent regulatory patterns as basic constituents of the network. Initial studies first found the presence of the simplest of these patterns, the one-element feedback loop [3]. More exhaustive examinations confirmed the prevalence of these structures [approximately 56% of *E.coli*'s TFs are autoregulated, Materials and methods], and further observed the use of other types of regulatory circuits, generally termed as network motifs [4].

What do motifs emerge? Two general models are currently considered. The most accepted one associates the presence of motifs to the singular information-processing tasks they can accomplish (see [5], for a review). Additional properties could further support this picture, such as the strong dynamical stability exhibited by motifs [6], or the correlation of their abundance with the global functional requirements acting on the network, e.g., the necessity of short response times in transcription [7]. Motifs in this model are then adaptive and isolated working units, a view that seems partially confirmed by their appearance in several transcriptional networks (e.g., those of *Bacillus subtilis* [8] or *Saccharomyces cerevisiae* [9]), and by the experimental confirmation of some of their suggested functional attributes [10], [11], [12], [13], [14].

An alternative model proposes that the occurrence of motifs is rather nonadaptive. Motifs might arise, according to this hypothesis, by the action of neutral population-based forces –like random genetic drift [15]– as the result of intrinsic mechanisms of genome evolution [16], [17], [18], or as a consequence of null network growth constraints [19]. These aspects would additionally suggest a fuzzy signal of motif conservation across species, a prediction that seems partially confirmed [20]. Moreover, this interpretation also challenges the relevance of motifs as separated functional entities [21].

Here we propose an integrative approach to understand the assembly of motifs that partially solves this controversy. This strategy is based on characterizing the relation between motif assembly and the capacity of their constituent TFs to integrate and transmit biochemical signals. We thus denote the capacity to integrate several environmental stimuli as sensing specificity (coarsely quantified with the presence/absence of upstream transcriptional regulation on a TF which could also be described as sensing ability), and the specificity of the TF to transmit signals as response specificity (this being quantified by the size of the corresponding regulon). We show how these measures –even as coarse as they are– are helpful to obtain a new picture of motif assembly.

In particular, while analysis of the first feature reveals an uneven distribution of autoregulation, the study of response specificity uncovers a linkage between the tendency of a TF to establish FFLs and its regulon size, with the first decreasing with the second. Investigating this pattern in detail, we identify several causes of motif emergence.

First, TFs with small regulons correspond to a class of FFLs caused by the hierarchical regulation of groups of operons, mostly associated to catabolite repression. In comparison, TFs with large regulons (hubs) leads to the assembly of both FFLs and bifans aggregates by coregulating third elements in combination with other hubs. Interestingly, most of this coregulatory signal appears to be neutral following a network null model, which in turn helps us to strengthen the adaptive nature of a complementary small group of such aggregates. Hubs also exhibit a complementary regulatory strategy, i.e., exclusive regulation. This induces the emergence of large single input modules (SIMs) structures, whose appearance is again partially neutral. The basic idea of network motifs was that a number of regulatory patterns appeared in extant networks much more often than in randomized ones [4], [22]. Our analysis ultimately shows how only a small subset of motifs, within each motif class, originates the statistical signature that helped unravel these structures in *E. coli*'s transcriptional network.

## Results and Discussion

### Specificity and autoregulation

In analyzing why autoregulation, the simplest motif, is such a pervasive regulatory attribute in *E. coli*'s network, we could be asking two complementary questions. We could first ask whether autoregulation is usually acting in combination with other transcriptional interactions. This strategy could enhance the interpretation of environmental states [22] by allowing the integration of several signals, i.e., the bacterial sensing specificity [23]. A second question would be whether the distribution of autoregulation relates to the specificity of the response. We followed here a simple network-based definition, and roughly quantified this specificity by the number of genes regulated by the TF (regulon size), e.g., small regulons indicating highly specific responses.

**Figure 1:**
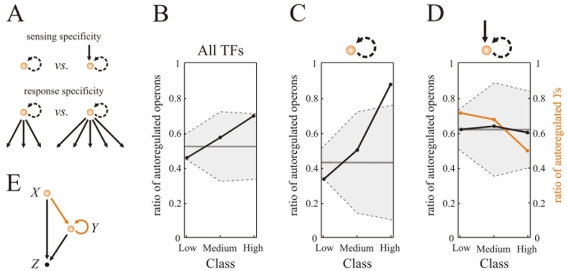
Distribution of autoregulation. (A) Sensing specificity (presence/absence of external regulation) and response specificity (regulon size). (B–D) Abundance of autoregulation in three TF response specificity classes quantified by regulon size. This includes first an analysis including all network TFs (B), followed by two more examinations considering those subsets of TFs without (C) or with (D) upstream transcriptional control, respectively. Classes: low (one to four regulated operons), medium (five to nine) or high (ten or more, considered as TF hubs). We also plotted the null behavior obtained by random sampling of the corresponding class –preserving group size– within the specific TF group (B,C, or D), 10 000 times (mean, continuous gray line, ±2 standard deviations, shaded area). Lines between points to help visualization. (E) Autoregulated TFs with upstream regulation as part of a FFL, a motif constituted by three elements *X*, *Y*, and *Z*, two of them being always a TF in this context (*X* and *Y*). In (D), the ratio of autoregulated *Y* for each specificity class is also showed (orange), see main text.

To study the first question, we partitioned all TFs in the network into two broad classes: TFs that do not experience any upstream regulation and those which do. Note that TFs of the first group are at the top of the network multi-layered structure [24], [25], [26], [27] –autoregulation, when present, would act in isolation– while those in the second class constitute the network lower layers. In this latter group autoregulation would act in combination with those TFs exerting upstream regulation (Fig. 1.A, top). We observed a smaller incidence of autoregulated TFs (ATFs) at the top (27 of 63 TFs are ATFs, 43%) as compared to lower layers (37/60, i.e., a 62% with *p* = 0.03 by assigning randomly all autoregulations, i.e., keeping fixed the number of ATFs and network hierarchy, 10 000 times, text S1 section 2).

To examine the second question, we introduced three TF classes in terms of response specificity (quantified by regulon size, Fig. 1.A, bottom). We observed that the tendency to be autoregulated grows with regulon size (Fig. 1.B). Moreover, both sense and response specificity could underlie selection for autoregulation. For instance, a large regulon size TF (a hub) at the top of the network could sense very general nutrient conditions and react by globally changing bacterial physiology [26], [27]. One could hypothesize that autoregulation in this case would contribute to a more precise control of the expression of the TF inducing such major physiological changes [22] (see also following discussions).

We thus studied sense and response specificity in combination. We found that the set of TFs lacking upstream regulation and with low regulon size are hardly autoregulated (Fig. 1.C). However, within this same set, hubs are mostly autoregulated (7 out of 8 hubs are ATF, e.g., CRP). These patterns are not observed in TFs under upstream control. In this case, operons exhibited a relatively homogeneous presence of autoregulation, independent of response specificity (Fig. 1.D).

### Autoregulation and the assembly of complex motifs

While a relation between autoregulation and regulon size (response specificity) was apparent in some of the previous patterns, the homogeneous distribution of ATFs in lower layers alternatively suggested a fairly neutral linkage between these properties. Could this distribution be masking some other patterns of network organization? Interestingly, ATFs with upstream regulation are common constituents of FFLs [5], with the ATF and the additional regulator as *Y* and *X* of this motif, respectively (Fig. 1.E), so we asked if this association could reveal any pattern.

To investigate this, we initially counted the number of FFLs with an *Y*-element belonging to each of the TF response-specificity classes, and within these groups the percentage of FFLs with autoregulated *Y*s. This revealed a striking dependence with specificity, ranging from a 71% of *Y*s autoregulated in the low regulon-size class to a 50% in the high class (Fig. 1.D, orange line).

**Figure 2:**
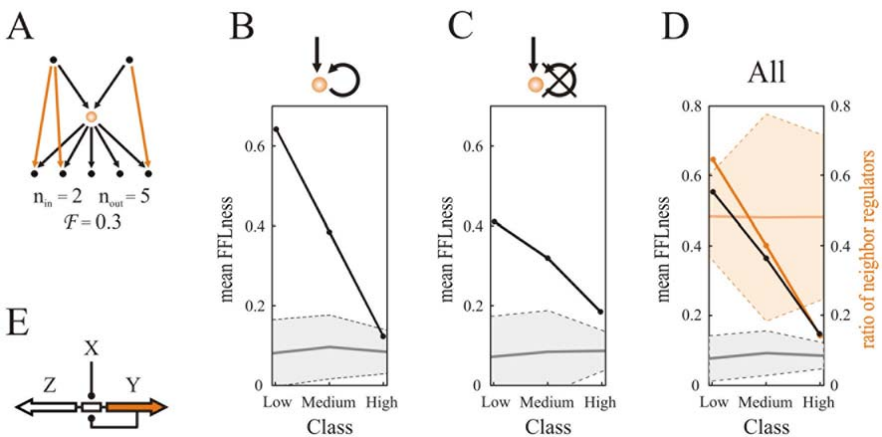
Assembly of FFLs and TF regulon size. (A) Computing FFLness: the maximum number of FFLs that can be potentially assembled by the TF in this example is *n*_in_ *n*_out_ = 10. Imagine that only 3 FFLs were actually observed (orange arrows), then this TF would have *F* = 0.3. (B–D) Mean FFLness as a function of regulon size for TFs with upstream control (classes defined as in Fig. 1). (B–C) autoregulated/non-autoregulated TFs. (D) all TFs with upstream control. The null behavior obtained in a network null model (Materials and methods; mean, continuous gray line, ±2 standard deviations, shaded area) is also plotted. In (D) we additionally showed the ratio of TFs –with upstream control– regulating a neighbor operon (dark orange) and its corresponding null (mean, continuous light orange line, ±2 standard deviations, orange-shaded area). Lines between points to help visualization. (E) Divergent architecture could promote FFL assembly. Spacer between two divergent operons (*Y* and *Z* in FFL) could include a binding site leading to the coregulations of these operons by an upstream TF (*X*) and by the autoregulated TF (*Y*). Note how this genomic architecture links autoregulation to neighbor regulation [30], [28], [29], [31].

To better understand this dependence, we quantified the tendency of a TF (autoregulated or not; but with upstream regulation) to establish FFLs with a measure that we named the FFLness (*F*, 0≤*F*≤1). This score is the ratio between the number of FFLs with this TF –as *Y*– found in *E.coli*'s network, and the maximum number of FFLs that such TF could potentially assemble (given by the product of the number of upstream TFs regulating *Y* and its regulon size, Fig. 2.A, and Text S1 section 2 and Figs. S1, S2, S3, S4). Figs. 2.B–C shows FFLness as a function of response specificity for autoregulated and non-autoregulated TFs, both in the extant network and in a null model (considering randomized networks with the same connectivity sequence, Materials and methods).

The distribution of autoregulation discussed before is confirmed in the new FFLness measure. For instance, the excess of autoregulation in the low regulon-size *Y*s (Fig. 1.D, orange line, low class) is also reflected in a stronger mean FFLness observed in the low class ATFs with respect to the non-autoregulated ones (compare mean FFLness values of low class in Fig. 2.B and Fig. 2.C, respectively). In addition, the FFLness measure highlighted a characteristic decay with regulon size in the establishment of FFLs by a TF, not found in the null model (Figs. 2.B–D, shaded areas). Since this decrease is observed in both autoregulated and non-autoregulated TFs, and since the distribution of autoregulation is, in contrast, homogeneous with regulon size (Fig. 1.D, black line), we asked what additional factors could help explain the strong tendency to establish FFLs (high FFLness) and its decay.

### What underlies the strong tendency to establish FFLs?

In the following, we considered two neutral models that could contribute to the strong tendency of low regulon-size TFs to establish FFLs: genomic architecture and homology between motif constituents. For the first model, we examined the association between this signal and neighbor regulation –of a TF on a genomically adjacent operon– a characteristic genome architecture in prokaryotic transcriptional control [28], [29] that can readily promote FFL assembly (Fig. 2.E). However, genome architecture could only partially explain high FFLness, as low regulon-size TFs with upstream regulation also showed a strong disposition to establish FFLs with nonadjacent operons (Figs. S1–S2) that did not even colocalize in the genome in broader terms (text S1 section 4). We thus analyzed if homology between motif components could be explaining this pattern [32], [33]. The fact that these combined models could not totally account for the strong FFLness score, and that we also identified a remarkable functional association between the constituents of these FFLs, indicates selection for a pattern of aggregated FFLs that we propose in a later section in detail.

#### Is genome architecture driving high FFLness?

In Fig. 2.D we plotted the proportion of neighbor regulation in TFs under upstream control. Low regulon-size TFs are indeed enriched by this architecture, a signal that decreases with regulon size (similar to FFLness, also in Fig. 2.D). This suggests neighbor regulation as an important factor underlying part of the high FFLness signature. Indeed, although autoregulation appeared linked to high FFLness (but not significantly, ATFs: *F* = 0.64; non-autoregulated TFs: *F* = 0.41, *p* = 0.12, Wilcoxon rank sum test; Figs. 2.B–C, low regulon-size class), neighbor regulation is a stronger determinant (TFs with adjacent regulation: *F* = 0.70; TFs without: *F* = 0.29, *p*<0.01, Wilcoxon rank sum test).

In this same analysis, we also recovered the connection between neighbor control and autoregulation –previously reported [30], [28], [29], [31]– for the TFs at the top of the network (*p* = 0.01, two-tail Fisher's exact test). This relation was lost in those TFs with upstream control (*p* = 0.5, Yates-corrected χ^2^-test, see also text S1 section 3 and Table S2). This could be partially caused by a failure to report autoregulation in some cases –which can be particularly difficult to resolve for divergent architectures [31]. Alternatively, the acquisition of new binding sites to enable the upstream control could in some occasions interfere with the autoregulatory binding site.

#### Is homology driving high FFLness?

We considered two possible models additionally contributing to the high FFLness score. The first one explained this tendency by the homology between those TFs encoded in the *X*- and *Y*-operons. Alternatively, a second model analyzed if nonadjacent *Z*s (nad*Z*s) inherited the same regulation of the central unit –which would lead to the establishment of additional FFLs– by duplication of genes belonging to such central set. This unit was defined as the group of genes constituted by the operon encoding the TF acting as *Y* and, when applicable, by those of its *Z*-operons adjacently located (which included also second adjacents, to control for tandem duplications).

**Figure 3:**
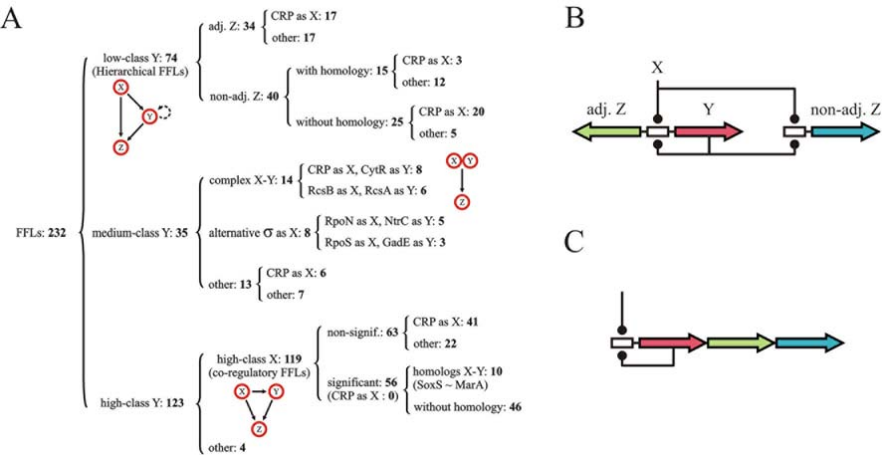
FFL classification. We divided the 232 FFLs identified in the network into three response specificity classes as defined in Fig. 1. i) Low class, hierarchical. These FFLs present *Y*-elements with small regulon size and a tendency for autoregulation. We divided this class in two subfamilies defined by whether *Y* regulates, or not, a genomically adjacent gene: 34 FFLs have an adjacent *Z*, while 40 FFLs do not have an adjacent *Z*. In the first subfamily we scored the number of FFLs with *X* being CRP. In the latter subfamily, we quantified those FFLs exhibiting homology and, again, the role of CRP in these groups. For instance, we found 20 low regulon-size FFLs with CRP as *X*, with *Z* not adjacently located and whose assembly does not follow the homology model. ii) Medium class, complexes. FFLs established with *Y*s of this class are enriched by pairs (*X*,*Y*) in which the action of one TF totally relies on the presence of its partner, e.g., RpoN on NtrC, see Fig. S3). iii) High class, hub coregulation. These FFLs mostly correspond to those motifs whose *Y*-elements are hubs. We again characterized this class with respect to homology and CRP influence. Note that all numbers indicate those FFLs found in each category, see also Table S1. (B–C) Dual regulatory logic in hierarchical FFLs (B) *vs.* polycistronic (C) designs, color code represents functionally equivalent genes, text for details.

We found 15 out of 40 FFLs constituted with nad*Z*s that could be explained with the homology models above (Fig. 3.A. and text S1 section 4). Thus, both null models only partially contributed to explain the strong tendency to assemble FFLs with nad*Z*s, even though we considered very permissive scenarios. For instance, our reasoning in the first model assumed that the duplication of *X* happened after this factor established its regulatory links, a relaxed assumption considering the prevalence of HGT [34] and the high rate of network rewiring in bacteria [35], while the conservation of regulation in the second model did not consider the influence in this conservation of the order of genes on the operons [36].

#### Is functional fine-tuning driving high FFLness?

What about the rest of FFLs that could not be explained by the models above? We observed a characteristic functional pattern based on the following features. First, in most cases CRP is acting as *X* of the FFL (20 out of 25 cases of FFL not explained by homology, Fig. 3.A). While this could be *a priori* expected due to the large regulon of CRP, we found that this role is particularly relevant in these 25 cases within the low regulon-size group (*p*<0.014, two-tail Fischer's exact test). Note that CRP is also dominant in the latter group as compared with the rest of FFLs (*p* = 0.008, Yates-corrected χ^2^-test).

**Figure 4:**
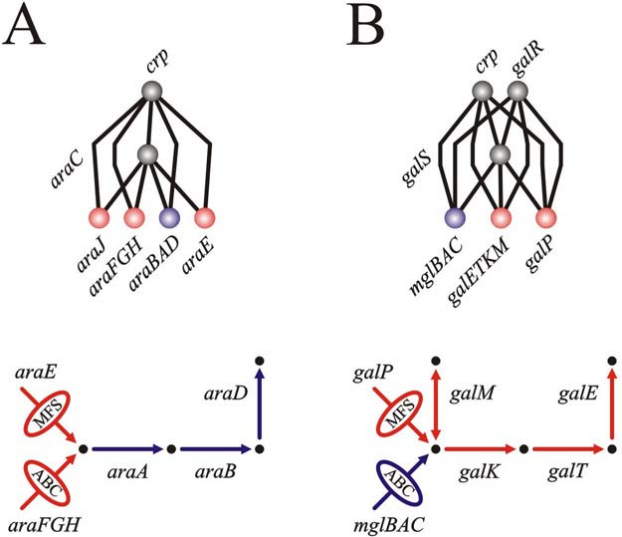
Examples of functional fine-tuning in *Z*s of hierarchical FFLs: the arabinose (A) and galactose (B) systems. In each case we plotted (top) the incoming/outgoing regulatory interactions associated to the TF sensing the specific sugar and those links involved in FFL assembly, and (bottom) the encoded metabolic pathway, with arrows or ellipses –crossed by arrows– denoting enzymes and transporters, respectively. Each system imports the corresponding metabolite by two different (non-homolog) transporter classes: MFS sugar/proton symporters and ABC transporters. MFS transporters encoded in *araE*, *galP* are homologs. ABC transporters in *araFGH*, *mglBAC* are also homologs. Note that both examples exhibited maximal FFLness, i.e., *F* = 1. Color code *Z*-elements: blue, adjacent *Z*; red, non-adjacent *Z*. The same color code applies to the encoded pathway steps. Text S1 for more examples and further discussions.

Second, the function of the genes encoded in nad*Z*s is remarkably related to the one exhibited by genes in the central unit. Sometimes genes in nad*Z*s and those encoded in the corresponding central operon are transporters responding to the same metabolite by alternative mechanisms. For example, arabinose and galactose sugars can be imported by low affinity proton-driven MFS symporters or by high affinity ATP-driven ABC transporters, Fig. 4. Other relationships do not necessarily imply different transporter classes. The transcriptional factor DgsA controls three nad*Z*s associated as follows: two of them encode sugar-specific components of respective glucose PTS transporters, while a third nad*Z* encodes the common non-sugar-specific components of this transporter type. Finally, those nad*Z*s encoding, apart from transporters, also enzymatic reactions are all associated to one-step pathways (e.g., *chbBCARFG* in charge of chitobiose degradation) which are complementary to those found in their respective central unit (text S1 section 4 and appendix, Table S10, for details).

### Hierarchical aggregated FFLs as adaptive functional units

What is the overall picture suggested by the discussions above? We emphasize here the hierarchical regulatory scheme that we identified, and propose an adaptive scenario for its emergence. This scheme combines the action of a general and specific TF following a hierarchical logic mostly linked to catabolite repression: when glucose is absent, CRP regulation (*X* in most of these FFLs, Fig. 3.A) activates a number of genes enabling the sensing (*Y*s in FFLs, usually autoregulated) and metabolizing (*Z*s, adjacently or not adjacently located) of alternative sugar sources. *Y* in these FFLs is thus subordinated to *X* activity, and the control of each group of operons (*Z*s) by the corresponding (*X*,*Y*) hierarchical regulatory logic presents this type of aggregated FFLs as fully-fledged independent functional units. Autoregulation in TFs encoding *Y* also implies that this logic applies to this very same TF, suggesting that its presence is not just an optional regulatory design, but rather a fundamental ingredient of transcriptional control. The implementation of this hierarchical control might not be necessarily restricted to FFLs (see below).

With respect to the adaptive/neutral forces leading to the assembly of these aggregates, we can envisage the following scenario. Initially, pairs of neighborly regulated genes –in divergent orientation– can be horizontally transferred to *E. coli*, and then acquire an additional regulation by a global regulator, mostly CRP [37], in their intergenic region. This leads indirectly to the assembly of a core FFL which could only be a neutral byproduct of the previous process (Fig. 2.E). This neutral picture of FFL assembly seems to be lost when we consider the other FFLs in the aggregate.

The functional characterization of nad*Z*s revealed a very close relation among all *Z*s, adjacent or not (see Fig. 4, text S1 section 4 and appendix), and indicates that the emergence of these aggregated FFLs could be a consequence of selection for the (*X*,*Y*) combinatorial logic, independent of genome location. This model could be further supported if some similarity of expression and/or evolutionary dynamics between adjacent and non-adjacent *Z*s were observed. The first one was experimentally reported in the arabinose system (adjacent –*araBAD*– and nonadjacent –*araFGH*– *Z*s [10]). In addition, we found that the averaged phylogenetic co-conservation of the pairs (*Y*,*Z*) in γ-proteobacteria was larger than expected by chance, and that the difference in this co-conservation for adjacent and nonadjacent *Z*s, studied independently, was non-significant (text S1 section 4).

Moreover, we propose in this context the similarity between hierarchical FFLs and autoregulated polycistrons. Genes acting as *Z*s in the former would be part of the polycistron in the latter and could also be subjected to a hierarchical logic (by an external global signal and the specific one associated to the polycistronic autoregulation, Fig. 3.B–C). This equivalence is based on the following observations. It is implied by the fact that the set of low regulon-size autoregulated operons, which do not regulate adjacent ones, is enriched with long polycistrons only when exhibiting external regulation (Tables S3, S4, S5). It is also suggested experimentally. One of the *Z*-elements of the *gal* system (Fig. 4.B) was showed to exhibit the very same response speed-up to that observed in a negatively autoregulated polycistron [13]. Indeed, the *Y*-element of this FFL –GalS– is negatively autoregulated, sharing thus the very same transcriptional logic of this *Z*-element. When to present a hierarchical FFL or an autoregulated polycistron regulatory architecture could be related to the specific mechanisms of network evolution (see text S1 section 4) [38], [37].

### What underlies the weak tendency to establish FFLs?

Resuming the analysis of the tendency of TFs to establish FFLs, beyond hierarchical FFLs (Fig. 2.D), we first observed that FFLs established with medium regulon-size *Y*s are enriched by pairs (*X*,*Y*) in which the action of one TF totally relies on the presence of its partner, e.g., RpoN on NtrC (see Fig. 3.A and Fig. S3), in order to induce the expression of third operons (*Z*s) and of *Y* itself (which is always autoregulated in these cases). This directly leads to the emergence of a FFL structure. What about the drastic decay of FFLness found in TFs with large regulon size?

The decrease in FFLness in these TFs (autoregulated or not) made this signal closer to the one seen in the null model (although remaining statistically significant, Fig. 2.D). Such low value does not imply that these TFs do not establish FFLs –in fact more than half of the FFLs of the network has a hub as *Y*-element, Fig. 3.A– rather that a small fraction of their potential FFLs are assembled. Is then this family of FFLs mainly originated by neutral forces?

The likelihood of neutral assembly of a FFL by a given TF acting as *Y* (FFL_null_) is given by the product of the neutral FFLness (according to the network null model, i.e., *F*_null_≈.08, Fig. 2.D), the number of external regulations (*n*_in_) and the regulon size (*n*_out_): FFL_null_ = *F*_null_ *n*_in_ *n*_out_. This directly indicates that the null assembly (FFL_null_) scales with regulon size, and also that the low FFLness of *Y* hubs can still be associated to the appearance of a considerable number of FFLs. This is caused by the partial random overlap of the regulons of the potential *X* and *Y* TFs, an overlap favored when both TFs are hubs. Indeed, *X* elements are mostly hubs in the extant network (211 cases of the total of 232; in particular 119 FFLs have both *X* and *Y* hubs, Fig. 3.A). Is then neutral overlap a major contributing part of the FFLness score observed among hubs?

#### Hub combinatorial regulation, FFLs and bifans motifs

**Figure 5:**
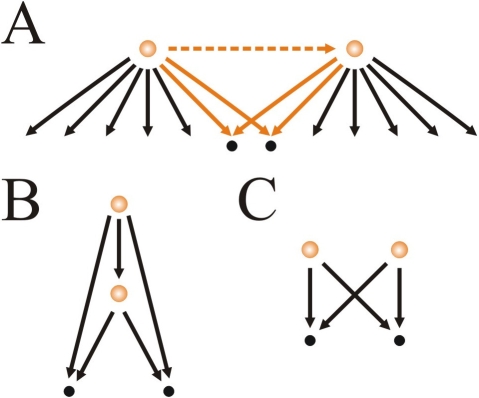
Motif assembly by random overlap of large regulons. (A) In this example a couple of hubs shares two target (corregulated) operons. As hubs may interact or not (dashed line), these coregulations lead to the assembly of aggregated FFLs (B) or bi-fan motifs (C).

We investigated the relevance of neutral hub coregulation as follows. We identified all possible pairs of hubs in *E.coli*'s network (23 hubs, 253 pairs). For each pair, we contrasted the coregulation observed in *E. coli* with a null averaged value obtained with randomized networks (Materials and methods and text S1 section 2). Interestingly, we found a fairly small number of significant coregulations after correcting for multiple testing (Table 1 and text S1 section 2). Note that the most significant ones correspond to five pairs in which one TF regulates the other, i.e., they are associated to FFL aggregates, while the remaining ones –when hubs do not interact– correspond to bifan aggregates [4], [39], [40] (Fig. 5).

**Table 1:** Pairs of hubs exhibiting significant coregulation.

TF pair		cre	cre_n_	*Z*-score^*^	FFL/Bi-fan^**^
SoxS	MarA	10	66.7	17.68	→
FliA	FlhDC	5	40.8	10.52	←
FNR	NarL	18	42.4	9.97	→
FNR	ArcA	16	27.3	5.21	→
RpoE	CpxR	7	21.4	4.74	←
IHF	RpoN	10	22.6	4.58	n.i.
FNR	IHF	18	25.9	4.39	n.i.
IHF	NarL	8	23.3	4.28	n.i.
IHF	Lrp	7	19.9	3.63	n.i.
CRP	ArcA	19	38.2	3.56	n.i.
CRP	RpoE	2	3.6	−3.21	n.i.

TFs in pairs sorted by regulon size. ^*^ Cutoff corresponds to adjusted *p*<0.05. ^**^ Arrows denote regulatory order. cre: number of coregulations, n.i.: non-interacting hub pairs. cre_n_: normalized cre as 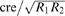, *R_i_* denotes regulon size. Note that the pair (CRP, RpoE) appears as the single case of significant anticoregulation (see main text).

What type of adaptive coregulations revealed this simple null? Gene duplication of regulatory hubs was suggested to play an important role in the assembly of FFLs in yeast [17], so we first analyzed if duplication could be contributing to these significant coregulations. We found that only the (MarA,SoxS) pair showed homology. This common regulation indeed arose by duplication [37]. Notably, in each of the rest of significant interacting pairs a coordinated way of action is particularly well documented. This is the case of the second top (FlhDC,FliA) pair, which is part of the genetic network controlling the temporal program of flagellar assembly, with FlhDC being its principal regulator, and FliA the flagellum-specific σ factor [12]. Additionally, the pairs (FNR,NarL) and (FNR,ArcA) regulate anaerobic respiration and fermentation. In this context, ArcA and NarL determine the type of respiration mode under the coordination of FNR [41]. Finally, RpoE is involved in heat shock and other stress responses [42]. This TF shows a strong tendency to exclusive regulation (40 out of its 51 regulatory outputs do not receive any other transcriptional regulation, see next section). However, reaction to membrane stress is coordinated by coregulation with CpxR, a constituent of the two-component regulatory system CpxA/CpxR, which senses this type of stresses (including misfolded proteins and degrading factors). Moreover, the set of non-significant coregulations (*p*>0.1, even before controlling for multiple testing) and those established by the (MarA,SoxS) pair implied a total of 73 out of 119 FFLs, with X and Y being interacting hubs, and whose presence could be explained by neutral processes alone according to the null network model considered.

It is also interesting to observe that the mean FFLness of the *Y*-elements being part of the significant FFLs (beyond MarA, i.e., FliA, NarL, ArcA and RpoE) is *F* = 0.34, while a much smaller averaged value was observed for the rest of hubs with upstream regulation and non-significant co-regulations, i.e., *F* = 0.07. This thus helped explain why we found a small, but significant, FFLness in the large regulon-size class: this signal is a mixture of the non-significant and significant coregulations represented in the decayed, but yet significant, total score (Fig. 2.D).

What about those significant coregulations established by non-interacting hubs in Table 1? The integration host factor (IHF) regulator appeared recurrently in this case (4 out 5 cases). This trend could be explained by the intrinsic architectural role of IHF that facilitates the action of other TFs by controlling DNA bending [42]. Interestingly, although there are 11 pairs of homolog hub pairs without mutual regulation, none of them leaded to a significant coregulation. For example, CRP and FNR –the hubs with the largest regulons– are homologs [27], and coregulated a number of operons similar to the null value (observed: 24 operons, random: ∼20, *Z*–score = 1.15). The pair (CRP, RpoE) did arise as a single case of significant anti-coregulation, i.e., they coregulated less operons than expected by chance. The autonomy of the RpoE stress response is thus reflected in a necessary uncoupling of the metabolic context.

Since duplication of hubs did not play a relevant role in these significant coregulations [17], we asked if duplications of coregulated genes could contribute to this signal. This could be partially the case in the coregulations established by IHF, FNR and NarL (these hubs shared 8 coregulated operons), and only under a permissive criterion (inheritance of binding sites imposes more strict constrains to the location of homolog genes in their respective operons [36]). However, there are functional arguments for the convergent establishment of these coregulations, since IHF enhances the action of the activators NarL and FNR, as stated above [43].

### Exclusive regulation and single input modules

A somehow complementary regulatory strategy to combinatorial regulation is linked to exclusive regulation, this term referred to the absence of any additional regulation on a group of operons, beyond that of a given master TF. We first investigated if exclusive regulation is significantly observed in the extant network (comparing with a null network model, as previously). Note that this question is equivalent to ask whether single input modules (SIMs) are network motifs [4]. We found 27 exclusive regulations (≥3 target operons) or SIMs in *E.coli*'s network. This number is not significantly different to the random score (30.5 SIMs, *p* = 0.11). However, the mean number of target operons per SIM is indeed larger than expected (observed: 10.3 targets, expected: 8.4, *p* = 0.0032), confirming that large SIM structures are network motifs. Could this exclusive regulation be a statistically significant pattern uniquely associated to a small number of SIMs as we found in the case of the hub coregulation signal?

To analyze SIM motifs individually, we computed for each TFs in the network regulating≥3 operons the ratio between the number of operons controlled exclusively and its regulon size –all interactions with same sign, dual regulations not considered. We named this score the SIMness (*S*, 0≤*S*≤1) of the TF, and compared it to the averaged value obtained in a null network model (text S1 section 2). We discovered only a limited number of large regulon-size TFs with significantly high SIMness (Table 2). These structures are undeniably among the most isolated functional units in the transcriptional regulatory network.

**Table 2:** Positive and negative SIMness.

TF	*R* ^+^	*S* ^+^	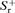	*Z*-score*
RpoE	51	0.78	0.33	7.34
CRP	117	0.15	0.38	−5.98
Fis	41	0.68	0.33	5.10
RpoH	25	0.68	0.31	4.07
IHF	28	0.07	0.33	−3.01
TF	*R* ^−^	*S* ^−^	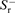	*Z*-score*
LexA	19	0.89	0.31	5.65
Fur	28	0.57	0.31	3.20

* Cutoff corresponds to adjusted *p*<0.05. *R^i^*: regulon size, *S^i^*: SIMness score, 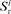: random SIMness score, *i*: positive or negative.

What functions are associated to these SIMs? They generally corresponded to autonomous systems able to rapidly induce urgent cellular responses. The SIM for which Fis is the master (positive) regulator is constituted by 28 operons involving a total of 75 genes. These genes are mostly constituted by transfer or ribosomal RNA genes (70 out of 75) coordinately expressed as adaptation to rapid growth conditions [44]. Three of the remaining cases are stress response regulators: LexA, exhibiting the highest (negative) SIMness, controlling DNA damage response [14], RpoE and RpoH, regulating several stresses like those related to heat shock [42] (RpoE also showed a strong coregulation when acting with CpxR, as discussed before). Finally, Fur is in charge of the control of iron homeostasis [45]. Homology is again not relevant in these significant SIMs, like in the case of significant hub coregulations. We only found one case of homology between the master regulator and its targets (LexA,UmuD). Homology among target genes was also rare (data not shown).

Note that Table 2 also included two TFs which displayed significant anti-SIMness, i.e., they regulated exclusively less operons than expected by chance. Anti-SIMness of CRP and IHF are a consequence of their strong bias to coregulation. We argued above that IHF is involved in the assembly of bifans in combination with several hubs. Equivalently, we found that CRP is associated to a combinatorial logic of global and specific metabolic signals, in coordination with low regulon-size TFs.

### Conclusions

What type of questions should we ask in addressing the causes of the emergence of network motifs? Here, we initially focus on two measures of functional specificity of *E. coli*'s TFs based on their corresponding in/out network degree [1], [22]. While this association is surely very coarse, it helps us nevertheless to identify two patterns linked to the network simplest motif, i.e., autoregulation. First, TFs with large regulons at the top of the network hierarchy are mostly autoregulated (Fig. 1.C), even though there is a small incidence of this feature in TFs of this layer. This should not be necessarily a surprise, since such global TFs at the top of the hierarchy can elicit a considerable change in bacterial physiology [25], [26]. In such scenario, autoregulation not only contributes to the precise integration of environmental states, but can also avoid noisy fluctuations of TF expression [5]. For instance, the autoregulatory circuit of the *crp* gene, one of the TFs at the top of the network hierarchy, plays a major role in CRP signal integration [46], while LexA autoregulation (another top global TF) prevents false (noisy) triggering of the SOS response in *E. coli*, due to transient fluctuations in the inducing signal [14].

A second, and more difficult pattern to interpret, is found in TFs with external transcriptional regulation. In this case, the distribution of autoregulation appears independent of response specificity, i.e., regulon size (Fig. 1.D). Notably, when we quantify the linkage between these TFs and the assembly of more complex motifs (specifically, its role as *Y* element of a FFL or FFLness, Fig. 2), this reveals a strong dependence between response and motif appearance. TFs enabling specific responses (small regulon size) tend to establish relatively more FFLs with their regulated genes than those inducing less specific responses (large regulon size; the decay of FFLness with regulon size is generally observed, i.e., even when specificity classes are not explicitly considered, data not shown).

That low regulon-size TFs –with upstream transcriptional regulation– tend to constitute FFLs with most of their regulon could be ascribed to several neutral constraints, and we analyze the two *a priori* more direct ones, i.e., genome architecture and homology of the constituents of the FFL. Are these factors fully explaining this tendency? The answer appears to be no. Indeed, a careful functional analysis of this family of FFLs highlights a hierarchical logic mostly linked to catabolite repression (Fig. 3.A). This logic is also found in autoregulated polycistrons which points at stronger selective forces acting on this type of regulation than on the specific genetic implementation. Which forces ultimately determine either architecture is hard to tell. In summary, the previous reasoning presents this class of aggregated FFLs as isolated working units beyond those arguments relying uniquely on statistical overrepresentation (see below).

The analysis of the linkage between TF response specificity and FFL emergence provides another relevant pattern. This is the drastic decay in the tendency to assemble FFLs when the *Y* element is a hub regulator. Since the neutral likelihood of establishing a FFL scales with regulon size, we ask to what extend those FFLs with *Y*s being hubs were mostly nonadaptive.

To answer this, and considering that most *X*s in the extant network are hubs, we contrast the coregulation between hubs observed in *E. coli* with the averaged value obtained in a null network model. We find that only a small set of coregulations appear significant under this null (Table 1), but exclude one of them – (SoxS, MarA)– as adaptive since it exhibits duplication. Interestingly, the rest of potentially adaptive coregulations lead to a number of FFL and bifan aggregates with remarkable function coordination, e.g., (FliA, FlhDC) related to flagellar control or several bifans associated to IHF, the integrator host factor regulator. Finally, by investigating a complementary strategy to coregulation, i.e., exclusive regulation, and contrasting it again to a network null, we identify a small group of SIMs –mostly stress response systems (Table 2)– that could also be putatively considered as adaptive, e.g., the LexA DNA damage response SIM.

Overall, the results presented here help to better understand the explicit functional signature behind the statistical definition of network motifs in *E. coli*. These motifs were originally recognized as patterns recurrently found in the extant transcriptional network when compared to a degree-preserving random one. This was done by using a summarized statistical score linked to any considered circuit architecture, i.e., counting the number of regulatory patterns of any particular type and comparing it with the null value. This work used the same kind of null-hypothesis model to show that not all constituents of a given motif class are equally unexpected. We also argued that those that appeared adaptive (like the FFLs with low regulon-size *Y*) could be subjected to other selective forces not necessarily linked to the computational tasks associated to the motif. For the rest of motifs appearing neutral, it is difficult to reject them a priori as adaptive units, as some sort of selection to maintain these edges in the network should be at work (e.g., [5]). Moreover, from the global statistical overrepresentation arguments that led to the description of the network motifs, and even assuming that each of the regulatory links has been selected, one cannot deduce that each of the extant motifs is under selection as a functional entity. Thus, this work sharpens the original counting arguments and contributes to the observations that more elaborated neutral models (see, [15], [17], [19], [7], [47]) are required to fully understand the adaptive dynamics of biological networks.

## Materials and Methods

### Network data

We assembled a transcriptional regulatory network (TRN) with data from *Escherichia coli*'s RegulonDB (v.5.6) [2]. In this network, each interaction is given by the operon encoding the transcriptional factor (TF), that encoding the target gene/s, and a directional link (edge) representing the transcriptional regulation, being this positive, negative or dual (two links of unknown sign were also considered). We did not include those interactions based only on microarrays or undocumented experiments. The TRN is constituted by 681 nodes and 1109 edges between different nodes. 135 of the nodes are TFs, including alternative σ-factors. Within the TF nodes, there are 76 which are autoregulated (approximately 56%), with 12 of them showing no further regulation over any other operon (exclusive autoregulators). The TRN is available in our website (http://www.cnb.csic.es/~jpoyatos. Files *operon_names.txt* and *interactions.txt*, including operon list and specific interactions, respectively).

For additional considerations on the assembly of this TRN see text S1 section 1. We examined several features of this network and that assembled in [4], where the concept of network motifs was originally introduced (Tables S6, S7, S8, S9).

### Network null model

We used a null model based on [48], i.e., fixing the number/type of incoming and outgoing edges in the random network to those of *E. coli*'s. The randomization protocol exchanges two randomly chosen connections of the extant network, when both edges are of the same interaction type (*A*→*B*,*C*→*D* to *A*→*D*,*C*→*B*). This procedure is repeated twice the number of edges (2×1109) in order to obtain a fully randomized network (two links of unknown sign were considered as dual ones in these randomizations). This null effectively implies that TF binding sites emerge neutrally. Other statistical methods in text S1 section 2.

## Supporting Information

Text S1 Additional analysis and appendix.
(1.82 MB PDF)

Table S1 Classification of the 230 FFLs in the network based on the connectivity of their respective X- and Y -TFs. LC, MC and HC for low-, medium- and highconnectivity classes, respectively. We also distinguished between autoregulated (curved arrow) and non-autoregulated (crossed-curved arrow) TFs, and those belonging to first (1st-L) and lower-layers (low-L). Small numbers denote number of instances in each subgroup (TFs only regulating their own operon are not considered; Y -elements belong to lower layers of the transcriptional network). The use of the “central unit” association implies an alternative classification of FFLs based on the number of *nonadjacent* regulated operons. Following this criterion, *exuR*, *nagBACD* and *malT*, all regulating one adjacent operon and four nonadjacent ones, are considered low connectivity operons. The minor differences introduced by this latter classification -which is the one used in Fig. 2.A, main text- are enclosed in parentheses.
(0.01 MB PDF)

Table S2 Relative orientation between upstream/downstream adjacent genes (→) and TRN operons (⇒). Upstream divergent orientation (←⇒) is particularly enriched. Curved arrow, operons encoding an autoregulated TF; crossed-curved arrow, operons encoding a non-autoregulated TF; *low* curved arrow, operons encoding an autoregulated low-connectivity TF; *low* crossed-curved arrow, operons encoding a non-autoregulated low-connectivity TF; *not*(*low* curved arrow), operons encoding a TF of the TRN excluding autoregulated low-connectivity ones.
(0.01 MB PDF)

Table S3 First-layer AOs. LC, MC and HC for low-, medium- and high-connectivity classes respectively. In LC without adjacent regulation we distinguish the cases of polycistronic and monocistronic AOs. † d, divergent; u, unidirectional. ‡ Regulated second neighbors included. Calculations based only on microarray data enclosed in brackets. Ψ In those cases with adjacent regulation, we showed number of promoters corresponding to the autoregulated and the adjacent operon, respectively.
(0.01 MB PDF)

Table S4 Lower-layers AOs of low-connectivity class. When there is not adjacent regulation we distinguish the cases of polycistronic and monocistronic AOs. † d, divergent; c, convergent; u, unidirectional. In the *rhaSR* case there is adjacent regulation over both the upstream and downstream neighbors. ‡ Regulated second neighbors included. Calculations based only on microarray data enclosed in brackets. Ψ In those cases with adjacent regulation, we showed number of promoters corresponding to the autoregulated and the adjacent operon, respectively.
(0.01 MB PDF)

Table S5 Lower-layers AOs of medium- (MC) and high-connectivity (HC) classes. † d, divergent; u, unidirectional. ‡ Regulated second neighbors included. Calculations based only on microarray data enclosed in brackets. Ψ In those cases with adjacent regulation, we showed number of promoters corresponding to the autoregulated and the adjacent operon, respectively. ¶ *cmk-rpsA-ihfB* and *thrS-infC-rpmI-rplT-pheMST-ihfA*, encoding the two components of the transcription factor IHF, counted as a single node in the network (see the first section of text S1).
(0.01 MB PDF)

Table S6 General features of SO and CP networks. Curved arrow, operons encoding an autoregulated TF (autoregulated operons); crossed-curved arrow, operons encoding a non-autoregulated TF. Operons encoding a TF that only regulates its own operon in parentheses.
(0.01 MB PDF)

Table S7 Comparison between autoregulated operons in SO and CP networks. An autoregulated operon in the CP network can be autoregulated (curved arrow), non-autoregulated (crossed-curved arrow) or absent (Abs) in the SO network, and conversely. We specified those operons located in first and lower network layers. Operons appearing in the network only as target operons in parentheses.
(0.01 MB PDF)

Table S8 Coherent and incoherent FFLs in SO and CP networks (as defined in ref. [2], text S1). Coh: coherent FFLs; Inc: incoherent FFLs, Other: FFLs with at least one dual-type interaction (see also note 3 in text S1).
(0.00 MB PDF)

Table S9 Distribution of operons per layer in SO and CP networks. We showed explicitely the distribution of autoregulated (curved arrow) and non-autoregulated TF (crossed-curved arrow). † The two components of the *marRAB-rob* loop are considered to be located both in the 6th layer.
(0.01 MB PDF)

Table S10 Characterization of low-connectivity Y -TFs establishing FFLs with at least one nadZ. First and second columns: Y and X TFs -homolog pairs in bold (two-component systems are also shown). Third and fourth columns: functional characterization of proteins in the central unit and corresponding nadZs labeled with numbers. This also shows the homology relationship -highlighted by same color- between genes in nadZs and those in the associated central unit. Abbreviations: TF, transcriptional factor; 2c, two-component system; E, Enzyme; T, transporter; PTAE, periplasmic transportassociated enzyme; U, uncharacterized protein; NP, near pathway, products acting in regions of the metabolic pathway near those of the central unit; RP: redundant pathway, including proteins which constitute multienzymatic complexes with those encoded in the central unit; P: pathway, sometimes there is no pathway encoded in the central unit, but in the nadZs. See Appendix in text S1 for further details.
(0.00 MB PDF)

Figure S1 Regulatory links associated to lower-layers operons encoding a low-connectivity autoregulated TF (1≤out-degree<5). We showed incoming and outgoing regulations and also those additional ones to describe FFLs (X-Z interactions). Edges color code: blue, activation; red, repression; gray, dual regulation. Z-operons filling color code: black, Z- and Y -operon are adjacent; gray, Z and Y are second neighbors; white, Z and Y are not adjacent. Dashed lines denote links where the TF encoded in the autoregulated operon is not affected by the regulation. This particularly applies to the regulation of *pdhR-aceEFlpdA* by *arcA*, and leads to the constitution of two pseudo-FFLs. Abbreviations: *<rpo>*, *nlpD-rpoS*; *<hyp>*, *hypABCDE-fhlA*; *<hyc>*, *hycABCDEFGHI*; *<hyf>*, *hyfABCDEFGHIJR-focB*; *<rpoN>*, *lptB-rpoN-yhbH-ptsN-yhbJ-npr*; *<ihf>*, *cmkrpsA-ihfB*; *<csiD>*, *csiD-ygaF-gabDTP*; *<bae>*, *mdtABCD-baeSR*; *<pdhR>*, *pdhRaceEF- lpdA*; *<srl>*, *srlAEBD-gutM-srlR-gutQ*; *<tdcA>*, *tdcABCDEFG*. Averaged FFLness: <*F*> = 0.77.
(0.02 MB PDF)

Figure S2 Regulatory links associated to lower-layers operons encoding a low connectivity non-autoregulated TF (out-degree<5). Abbreviations: *<ompR>*, *ompR-envZ*; *<yiaK>*, *yiaKLMNO-lyxK-sgbHUE*, rest of abbreviations as before. Color coding as in Figure S1. <*F*> = 0.46.
(0.02 MB PDF)

Figure S3 Regulatory links associated to lower-layers operons encoding a medium connectivity autoregulated TF (5≤out-degree<10). In the alternative classification of TFs based on the number of nonadjacent regulated operons *nagBACD* is considered a low-connectivity operon. Maximal FFLness of *rcsA*, *glnALG* and *cytR* corresponds to pairs (X,Y) in which the action of one TF totally relies on the presence of its partner (RcsA on RcsB, RpoN on NtrC -encoded in glnG- and CytR on CRP). Abbreviations: *<mraZ>*, *mraZW-ftsLI-murEF-mraYmurD- ftsW-murGC-ddlB-ftsQAZ*; *<wza>*, *wza-wzb-wzc-wcaAB*; *<mutY>*, *mutYyggX- mltC-nupG*, rest of abbreviations as before. Color coding as in Figure S1. <*F*> = 0.66.
(0.02 MB PDF)

Figure S4 Regulatory links associated to lower-layers operons encoding a medium connectivity non-autoregulated TF (5≤out-degree<10). In the alternative classification of TFs based on the number of nonadjacent regulated operons malT is considered a low-connectivity operon. The type of transcriptional interaction between *cmk-rpsA-ihfB* and *flhDC* is not known (in black). Abbreviations: *<malK>*, *malK-lamB-malM*, rest of abbreviations as before. Color coding as in Figure S1. <*F*> = 0.39.
(0.01 MB PDF)
