# Supplementary material for: What Determines the Assembly of Transcriptional Network Motifs in Escherichia coli?
Source: PLoS One. 2008 Nov 6;3(11):e3657. doi: 10.1371/journal.pone.0003657 (PMC2577066; doi:10.1371/journal.pone.0003657)
Supplement: Text S1 — Additional analysis and appendix. (1.82 MB PDF) [file pone.0003657.s001.pdf]

Supplement

The determinants of transcriptional network motifs assembly in *Escherichia coli*

Francisco M. Camas and Juan F. Poyatos.
Spanish National Biotechnology Centre, Consejo Superior de Investigaciones Científicas (CSIC), 28049 Madrid, Spain.

Transcriptional network

We assembled a transcriptional regulatory network (TRN) with data from *Escherichia coli*'s RegulonDB (v.5.6) [1]. In this network, each interaction is given by the operon encoding the transcriptional factor (TF), that encoding the target gene, and a directional link (edge) representing the transcriptional regulation, being this positive, negative or dual. The TRN is constituted by 681 nodes and 1109 edges between different nodes. 135 of the nodes are TFs, including alternative σ-factors. Within the TF nodes, there are 76 which are autoregulated, with 12 of them showing no further regulation (the TRN is available in our website) [1].

Some additional considerations on the assembly of this TRN are

Heterodimers as single nodes. The IhfA and IhfB constituents of the heterodimer regulator IHF are encoded in two different operons, *thrS-infC-rpmI-rplT-pheMST-ihfA* and *cmk-rpsA-ihfB*, respectively. Because of their similar genomic architecture and regulation (IhfA and IhfB always work as heterodimer and are both under the same regulation), these operons are represented by a single node in the TRN. Similar reasoning applies to the HupA and HupB TFs, components of the heterodimer HU.

Heterodimers as two nodes. The heterodimer RcsAB, whose corresponding operons encode RcsA and RcsB, does not show the previous behavior. RcsB works independently as a homodimer activator (member of the 2-component system RcsC/RcsB). Moreover, RcsAB regulates *rcsA* but not *rcsB*. We thus considered *rcsA* as an autoregulated operon, with the assistance of the protein RcsB, and the operons encoding RcsA and RcsB as two nodes in the TRN.

---

[1] http://www.cnb.csic.es/∼jpoyatos. Files *operon_names.txt* and *interactions.txt*, including operon list and specific interactions, respectively. Here, we considered two links of unknown sign, but did not include those interactions based only on microarrays or undocumented experiments.

gntRKU operon. We interpreted *gntRKU* as two separated operons (*gntR* and *gntKU*). This prevents the pseudo-autoregulation of *gntKU* by a constitutive GntR, in which GntR would not regulate itself. This also impedes IdnR regulation over *gntKU* (but not over *gntR*) to establish a "pseudo-loop" (or non-dynamical loop) between *gntRKU* and *idnDOTR*.

Feed-forward loop motifs

We identified 230 feed-forward loops (FFLs) in the TRN (Table S1, and Figure 2.A, main text) [2]. We did not consider in this list two instances of "pseudo-FFLs". By this we refer to those motifs in which the gene encoding the Y-TF is not part of any transcription unit (TU) regulated by the X-TF (recall that in a FFL $X \rightarrow Y$, $Y \rightarrow Z$, and $X \rightarrow Z$). In both cases *arcA* and *pdhR-aceEF-lpdA* were not considered as X- and Y-element of a FFL, respectively, because ArcA only regulates the TU constituted by the *lpdA* gene, which is not including the putative Y-element of these FFLs, i.e., PdhR [3].

Transcriptional feedback loops

A recent study documented four transcriptional feedback loops –with more than one component– in *Escherichia coli*'s transcriptional network [3]. In our TRN only one of these loops remains. Why is this so? One missing loop is the previously mentioned case of non-dynamical loop constituted by *gntRKU*/*idnDOTR*. The other two missing loops appeared when regulations based only on microarray data were considered, and thus they did not occur in our TRN. The only loop that we did find was that constituted by the *marRAB* and *rob* operon pair (Figure S4).

Comparison between Shen-Orr *et al.*, and Camas and Poyatos transcriptional networks

We examined several features of our TRN (CP network) and that assembled in [4] (SO network), where the concept of network motifs was originally introduced.

[2] As a complement to these tables we included Figures S1-S4. These figures show the incoming and outgoing regulations of low/medium connectivity Y-operons. We showed also the additional links that constitute FFLs.

[3] A list with the 230 FFLs and the 2 pseudo-FFLs can be found in our website, http://www.cnb.csic.es/∼jpoyatos. The file *all_FFL.txt* contains the X-, Y- and Z-operons listed in the first three columns. We added a fourth column with the FFL class as defined in [2].

This includes comparisons on 1) network main properties (Table S6), 2) number of autoregulated operons (Table S7), 3) FFLs (Table S8), and 4) distribution of operons in the network multilayered structure (Table S9).

Main statistical procedures

FDR. We controlled the False Discovery Rate in situations of multiple testing, i.e., when several p-values are calculated simultaneously. We used the following procedure [5]: let $p_1 \leq p_2 \leq \ldots \leq p_m$ be a set of (ordered) unadjusted p-values, the corresponding adjusted p-values are computed as $\tilde{p}_j = min_{k=1,\ldots,m} \left\{ min \left(\frac{m}{k}\, p_k, 1 \right) \right\}, j = 1, \ldots, m$.

Randomized networks. We used a null model based on [6], i.e., fixing the number/type of incoming and outgoing edges in the random network to those of *E. coli*'s. The randomization protocol exchanges two randomly chosen connections of the extant network, when both edges are of the same interaction type ($A \rightarrow B, C \rightarrow D$ to $A \rightarrow D, C \rightarrow B$). This procedure is repeated twice the number of edges (2×1109) in order to obtain a fully randomized network. This null effectively implies that TF binding sites emerge neutrally.

Autoregulation. We asked two questions related to the distribution of autoregulation in the TRN. First, we examined the location of autoregulated TFs in the network coarse layer organization (top level *vs.* rest of levels). We used a permutation test in which we maintained the layer distribution of the extant network but randomized the location of the autoregulated TFs. We then measured the number of autoregulations located in the first-layer of the permuted network and compared to the observed value. The presence of autoregulated TFs in the first layer is smaller than expected ($p = 0.0104$, 10000 randomizations). Second, we analyzed how autoregulation correlated with regulon size, controlling for the network coarse layer architecture. We randomized the location of autoregulated TF within each layer and computed the number of autoregulated TFs found in the low-, medium- and high-connectivity classes. Only first-layer hubs showed a significant enrichment of autoregulated TFs ($p = 0.0135$, 10000 permutations). Alternatively, first-layer low-connectivity TFs exhibited a significant low rate of autoregulation ($p = 0.03$, 10000 permutations).

FFLness. We introduced in the main text FFLness ($\mathcal{F}$) as a measure applicable only to lower-layer TFs regulating ≥ 1 operon(s) without including autoregulation. For any of these TFs, $\mathcal{F}$ is the ratio of the number of its target genes being part of a FFL (with the corresponding lower-layer TF as Y) and its regulon size. We have thus a set of {lower-layer TF $\rightarrow$ target operon} pairs constituting (or not) a FFL (we labeled every pair as 1: constituting a FFL, or 0: no FFL). To examine the significance of the observed FFLness measures, we randomized all labels and computed the mean $\mathcal{F}$, controlling for connectivity class. We applied the same protocol controlling for connectivity and also presence (absence) of autoregulation. We found a significantly high $\mathcal{F}$ for low and medium class autoregulated TFs ($p < 10^{-5}$ in both cases, 100000 permutations). Autoregulated hubs, in contrast, exhibited lower $\mathcal{F}$ than expected ($p < 10^{-5}$, 100000 permutations). Non-autoregulated TFs did not exhibit these patterns (the use of the alternative class definition discussed in Table S1 showed similar qualitative results). Regulatory links associated to computation of $\mathcal{F}$ for low- and medium-connectivity TFs are plotted in Figures S1-S4.

FFLness within a connectivity class. For each connectivity class, we analyzed if the presence of autoregulation influenced $\mathcal{F}$. We measured the difference between the averaged $\mathcal{F}$ of autoregulated and non-autoregulated TFs ($\langle\mathcal{F}\rangle_{aut} - \langle\mathcal{F}\rangle_{nau}$). Only autoregulated low-connectivity TFs (regulating one to four operons) exhibited a larger $\mathcal{F}$ ($p = 0.02$, 10000 permutations). Autoregulation and adjacent regulation are tightly linked (see below). Since there are cases of adjacent regulation by non-autoregulated operons, and to control for unreported autoregulations –which can be particularly difficult to resolve for divergent architectures [7]– we compared low-connectivity low-layer TFs which regulate/do not regulate adjacent genes by measuring the difference $\langle\mathcal{F}\rangle_{adj} - \langle\mathcal{F}\rangle_{nad}$. TFs regulating an adjacent operon exhibited larger $\mathcal{F}$ independent of autoregulation ($\mathcal{F}$ of TFs regulating adjacent genes = 0.79, $\mathcal{F}$ of TFs not regulating adjacent genes = 0.4, $p < 0.01$, 10000 permutations).

Significant coregulations by hubs. We counted how many coregulations were established on average by each possible pair of hubs (23 hubs, 253 pairs) in 10000 randomized networks and compared it with those of the extant network. We obtained in this way a set of 253 unadjusted p-values that were corrected for multiple testing as described before.

Significant SIMs. SIM motifs correspond to TFs exclusively regulating ≥ 3 operons (under the same interaction type). There are 36 TFs acting as master regulators of positive SIMs and 35 TFs for negative ones. For each of these TFs we counted how many operons they regulate in a exclusive way in a set of 10000 randomizations and compared this random score with the one observed in *E.coli* (p-values of positive and negative SIMs were adjusted independently).

Genomic features of the autoregulated operons

Orientation of genes adjacent to the autoregulated operons. A divergent genomic architecture is linked to neighbor coregulation and to the potential assembly of FFLs [7–10]. Here, we specifically asked to what extent this divergent design is enriched in the TRN. Thus, for each of the 681 operons of the TRN, we considered the relative orientation of the upstream/downstream adjacent gene –note that these genes may or may not be part of the TRN– and compared it with a null (10000 randomizations of the absolute orientation of each operon, keeping fixed the number of operons encoded in each strand of the chromosome). Divergent orientations are particularly observed for upstream genes (Table S2). This bias is stronger in the subset of autoregulated operons, and still further enhanced in those with a low connectivity. This bias towards divergent orientation was not observed in non-autoregulated operons. Orientation of adjacent and downstream genes did not show any special bias.

Operon structure. We examined the polycistronic/monocistronic architecture of those AOs that, being part of the low connectivity class, do not regulate an adjacent operon. While there is no particular bias to either design in the first layer, polycistronic AOs are considerable enriched in the lower layer (top level: 3 monocistrons + 3 polycistrons, lower levels: 3 monocistrons + 12 polycistrons, Tables S3-S4). Among polycistrons associated to lower layer ATFs those exhibiting a low connectivity are on average the simplest in terms of TUs, even when they are large (Tables S4-S5). Additionally, 9 of these ATFs only regulate its own operon. Thus, low-connectivity ATFs of lower layers are linked both to the polycistronic design and the assembly of FFLs.

FFLs with a low-connectivity Y-operon

The definition of the "central unit" (see main text) applies to all lower-layers TFs of the low-connectivity class (34 operons, Figures S1-S2) and two additional operons (*nagBACD* and *malT*) –both regulating one adjacent operon and four nonadjacent ones, see comments in Table S1. 28 operons of this set are involved, as Y-elements, in the assembly of 72 FFLs (plus the two known pseudo-FFLs, see above). In addition, 53 different operons act as Z-elements of these FFLs. Approximately half of the previous Y-operons (15/28) regulate at least one nonadjacent Z-operon (second neighbors excluded, see Table S10 and the Appendix of this supplement). There exist 30 of such nonadjacent Zs (nad$Z$s), involving 28 different operons (with two cases of shared nad$Z$s: *galETKM* and *manXYZ*, acting as Z-operons of two different Y-operons). Finally, note that to identify homology, we compared amino-acid sequences by Blast with an E-value threshold of 10^{-10} (other threshold values did not change qualitatively our results).

Central unit - nad$Z$s homology. We searched for those nad$Z$s that encode at least one gene homolog to those of the central unit. We obtained 7 out of 30 nad$Z$s with such relationship (Table S10 and Appendix). This number is bigger than expected by chance ($p < 0.0001$ by randomly reassigning 10000 times the set of all nad$Z$s to the set of central units with the restrictions that i) the number of nad$Z$s regulated by the Y-operon of each central unit is fixed, ii) an operon is never assigned to itself [4], and iii) an operon is never assigned twice to the same block because of the mentioned shared Zs).

X-Y homology. The 15 Y-operons regulating nad$Z$s and their respective X-operons constitute 42 different (X,Y) pairs. We analyzed the homology between genes encoding the X and Y TFs, respectively. We found 6 cases of homolog pairs (Table S10 and Appendix), larger than expected by chance ($p = 0.0003$, by permuting 10000 times TFs and controlling the cases where an operon is paired with itself).[5]

FFLs without homologies. About two thirds (24/38) of the hierarchical FFLs constituted with nad$Z$s cannot be explained by homology-based models (Figure 2.A, main text). We observed that these nad$Z$s are enriched by operons only encoding transport related protein, and that they are under the con-

[4] This could be possible because in the extant network the operon *malT* is both a Y-operon –with four different Zs– and a Z-operon of the Y-operon *dgsA*.

[5] *gadW* is found in both X and Y roles.

trol of CRP. These transporters are functionally related to those transporters encoded in the corresponding central unit, yet they are not homologs. Is the transporter located in the central operon, and thus physically linked to the TF, anyway different to those placed nonadjacently? Homologies across transporters associated to different FFLs groups –a given central unit and its associated nad$\mathbb{Z}$s– allowed us to compare aspects of function and genomic location. Examples of these homologies are the MFS-symporters or ABC-transporters of arabinose and galactose, and also the glucose and the (very related) N-acetyl-D-glucosamine PTS uptake systems. We found equivalent functions encoded in adjacent or nonadjacent locations. For instance, while the MFS- and ABC-arabinose transporters are both encoded in nad$\mathbb{Z}$s, the galactose ABC transporter genes are located in the central unit. Additionally, unlike the glucose uptake system located in nad$\mathbb{Z}$s, one of the specific components of a N-acetyl-D-glucosamine PTS transporter is encoded in the central unit (see Appendix).

Hierarchical FFLs vs polycistronic strategies. We proposed in the main text how an adaptive model based on the establishment of a hierarchical logic on a small set of genes acts as a unifying determinant leading to the occurrence of both hierarchical FFLs and low-connectivity polycistrons in lower layers (Figs. 2.B-C –main text– and Tables S3-S4).

What aspects could influence the presence of either control strategy in a given context? Reasons for the separation in different operons of coregulated genes than act together in a metabolic pathway has been discussed [11]. In brief, this separation allows differential regulation of each operon (enabling temporal programs of gene expression). A polycistron architecture might not be considered, in this sense, an optimal solution as it could induce the production of some proteins –encoded in the polycistron– before needed. However, this latter strategy can favor the transference of the encoded enzymatic tools across species by horizontal gene transfer (HGT). Neighbor regulation appears in this context as an intermediate solution, combining differential control and capability for successful lateral transfer [6] . Indeed, a large frequency of these events have been recently reported for neighbor regulators [12]).

A prediction of the differential expression model [11] is that genes are ar-

[6]The architecture of divergently transcribed operons also reduces the cost of maintenance and replication of an additionally promoter region.

ranged such that those encoded on the same operon do not skip functional steps in the pathway. This is precisely what we found for genes distributed among the operons in the central unit and the nad$Z$s (see Appendix). Note however that this result could also be due to the mechanisms explaining how bacterial metabolic networks grow, i.e., by direct uptake of genes encoding products involved in peripheral reactions by HGT [13]. This correlates with the enrichment of nad$Z$s with genes associated to the first steps of peripheral metabolisms. Effectively, we found that, in the most unquestionable cases of non-neutral evolution, nad$Z$s only encode transport related products –also associated to HGT events [13]). Moreover, although there are also a few cases of enzymatic products associated to one-step pathways, only in one case the pathway is located downstream of that encoded in the central unit (Y-operon: *dcuSR*, see Appendix).

Genome distance between the central unit and the nad$Z$s. For each central unit, we computed the mean distance to its nad$Z$s and then averaged over all units. We then randomized the full set of nad$Z$s and scored distances as before. The average distance of nad$Z$s to the central unit was not particularly small, even when including second neighbors as nonadjacent operons ($p = 0.1$, 10000 randomizations). We also calculated the "across distance" between the coordinates of each central unit and its associated nad$Z$s with respect to the *oriC* region, as chromosomal periodicity of evolutionarily conserved gene pairs has been also recently discussed [14]. This measure did not show any significant pattern either.

Averaged co-conservation of Y- and Z-operons. We considered the phylogenetic conservation of genes involved in the Y/Z operons through 75 species of γ-proteobacteria. Conservation of a particular gene was determined by reciprocal best-hit with an E-value threshold of 10^{-10} (other threshold values did not change qualitatively our results). We quantified co-conservation of each Y-operon/Z-operon by first averaging the Jaccard index [7] of proximity J for all the possible pairs of genes $(y,\ z)/y \in Y,\ z \in Z$. We then determined the average value of J over the set of 30 pairs constituted by the nad$Z$s with their respective Y-operons, and also for the 10 pairs with adjacent Z-operons (ad$Z$s, including here the second neighbors). The av-

[7] This normalized index is a ratio of the number of species in which both genes coexist divided by the total number of species considered. As a reference, the mean value of J for pairs of genes belonging to the same operon is 0.64 (for this set of Y- and Z-operons).

erage co-conservation of the pairs $\{Y$, all associated Zs –adjacent or not–$\}$ was significantly larger than expected by randomly reassigning the set of Zs ($p < 10^{-3}$, 10000 permutations) [8] . Moreover, the difference on this averaged co-conservation for nad$Z$s (0.40) and ad$Z$s (0.43) was not significant under the permutation of the ad$Z$/nad$Z$ labels (p=0.32, 10000 times).

Functional characterization. We examined in the Appendix the functional properties of the proteins encoded in the group of 15 low-connectivity Y-operons regulating nad$Z$s (second neighbors excluded, see also Table S10) and all their associated Z-operons, using EcoCyc database [15]. In some cases, the proteins are members of protein complexes whose additional constituents are not encoded in this group. We nevertheless enclosed this information in parentheses.

We included a simple cartoon showing the location of these proteins in their associated metabolic pathways. We used arrows or ellipses crossed by arrows to denote enzymes and transporters, respectively. When an protein is encoded in the central unit, we colored the corresponding symbol in blue. We used red for proteins encoded in nad$Z$s, and gray for proteins encoded in other operons. Some protein complexes required two colors at the same time.

We also described the previously discussed gene homologies, i.e, those between the central unit and nad$Z$s and those between TFs acting as X- and Y-elements of the FFL. Furthermore, we showed for adjacent regulations the relative direction of transcription with respect to that of the Y-operon: (d), divergent; (u), unidirectional (convergent cases were not found). We also indicated when the ad$Z$ is a second neighbor. Abbreviations: Y-op, Y-operon; nad$Z$, nonadjacent Z-operon; ad$Z$, Z-operon adjacent to the Y-operon (including second neighbors).

[8] To avoid that the signal of large co-conservation were only caused by the adjacent Zs, we applied the same randomization protocol only over the set of nad$Z$s. We obtained again that the averaged co-conservation of the pairs $\{Y$, nad$Z$s$\}$ was significantly large (p=0.02, 10000 permutations).

References

[1] Salgado H, Gama-Castro S, Peralta-Gil M, Díaz-Peredo E, Sánchez-Solano F, Santos-Zavaleta A, Martínez-Flores I, Jiménez-Jacinto V, Bonavides-Martínez C, Segura-Salazar J, Martínez-Antonio A, Collado-Vides J (2006) *Nucleic Acids Res* 34:D394–D397.

[2] Mangan S, Alon U (2003) *Proc Natl Acad Sci USA* 100:11980–11985.

[3] Cosentino M, Jona P, Bassetti B, Isambert H (2007) *Proc Natl Acad Sci USA* 104:5516–5520.

[4] Shen-Orr SS, Milo R, Mangan S, Alon U (2002) *Nature Genet* 31:64–68.

[5] Benjamini Y, Hochberg Y (1995) *J R Statist Soc B* 57:289–300.

[6] Maslov S, Sneppen K (2002) *Science* 296:910–913.

[7] Kolesov G, Wunderlich Z, Laikova ON, Gelfand MS, Mirny LA (2007) *Proc Natl Acad Sci U S A* 104:13948–13953.

[8] Korbel JO, Jensen LJ, von Mering C, Bork P (2004) *Nat Biotechnol* 22:911–917.

[9] Warren PB, ten Wolde PR (2004) *J Mol Biol* 342:1379–390.

[10] Hershberg R, Yeger-Lotem E, Margalit H (2005) *Trends Genet* 21:138–142.

[11] Zaslaver A, Mayo A, Ronen M, Alon U (2006) *Phys Biol* 3:183–9.

[12] Price MN, Dehal PS, Arkin AP (2008) Horizontal gene transfer and the evolution of transcriptional regulation in *Escherichia coli*. *Genome Biology* 9:R4

[13] Pál C, Papp B, Lercher MJ (2005) *Nat Genet* 37:1372–1375.

[14] Wright MA, Kharchenko P, Church GM, Segrè D (2007) *Proc Natl Acad Sci USA* 104:10559–10564.

[15] Keseler IM, Collado-Vides J, Gama-Castro S, Ingraham J, Paley S, Paulsen IT, Peralta-Gil M, Karp PD (2005) *Nucleid Acids Res* 33:D334–D337.

APPENDIX

Autoregulated Y-operon

Y-op: *gadAX*
-*gadA*: enzyme, glutamate dependent acid resistance
-*gadX*: TF

nadZ: *gadBC*
-*gadB*: enzyme, glutamate dependent acid resistance
-*gadC*: APC-transporter (aminobutyrate antiporter)

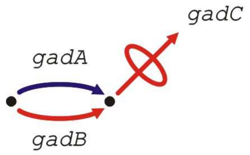

Notes:
- *gadA* and *gadB* are homologs
- GadX is homolog of the TF encoded in one of its four X-operons, *gadW*. These two operons are second neighbors only separated by the small gene *gadY*

Y-op: *mdtABCD-baeSR*
- *mdtABC* (+ *tolC*): RND-transporter. (drug exporter)
- *mdtD*: MFS-transp. (uncharacterized, drug efflux?)
- *baeSR*: 2-component system

nadZ: *acrD*
- *acrD* (+ *tolC* and *acrA*): RND-transporter (drug exporter)

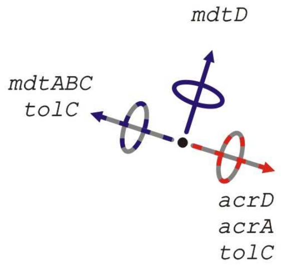

Notes:
- *mdtB*, *mdtC* and *acrD* are homologs
- *baeS* and *baeR* are homologs of the two-component-system genes *cpxA* and *cpxR* respectively; *cpxRA* is the only X-operon for *mdtABCD-baeSR*
- *tolC* encodes the common outer membrane component of several multidrug efflux systems

---

Y-op: *pdhR-aceEF-lpd*
- *pdhR*: TF
- *aceEF-lpd*: pyruvate dehydrogenase

nadZ: *lldPRD*
- *lldP*: LCT-transporter (lactate)
- *lldR*: TF
- *lldD*: lactate dehydrogenase

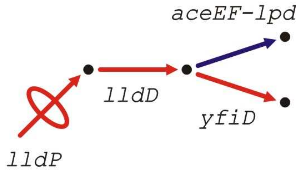

nadZ: *yfiD*
- *yfiD*: alternative stress induced pyruvate-formate lyase

Notes:
- *pdhR* and *lldR* are homologs
- *lldPRD* and *yfiD* are the respective Z-elements of the two pseudo-FFLs (see Fig. S1)
- *lldPRD* is an autoregulated operon

Y-op: *hypABCDE-fhlA*
- *hypABCDE*: proteins for maturation of hydrogenase
- *fhlA*: TF

adZ(d): *hycABCDEFGHI*
- *hycA*: uncharacterized
- *hycBCDEFG*: hydrogenase
- *hycHI*: protein for maturation of hydrogenase

nadZ: *hyfABCDEFGHIJR-focB*
- *hyfABCDEFGHIJ*: hydrogenase (putative)
- *hyfR*: TF
- *focB*: FNT-transporter (formate, putative)

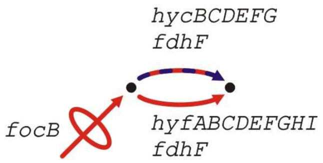

nadZ: *fdhF*
- *fdhF* (+ *hycBCDEFG*): formate-hydrogenlyase complex
- *fdhF* (+ *hyfABCDEFGHIJ*) : formate-hydrogenlyase complex (putative)

nadZ: *hydN-hypF*
- *hydN*: formate-dehydrogenase (putative)
- *hypF*: protein for maturation of hydrogenase

Notes:
- There are multiple homologies between the *hyc* and *hyf* genes
- *fhlA* and *hyfR* are homologs
- *hydN*, *hycB* and *hyfA* are homologs
- *hyfABCDEFGHIJR-focB* is an autoregulated operon

Y-op: *araC*
- *araC*: TF

adZ(d): *araBAD*
- *araBAD*: enzymes in arabinose degradation pathway

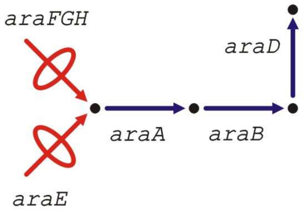

nadZ: *araE*
- *araE*: MFS-transporter (arabinose)

nadZ: *araFGH*
- *araFGH*: ABC-transporter (arabinose)

nadZ: *araJ*
- *araJ*: MFS-transporter (uncharacterized, sugar efflux?)

Y-op: *galS*
- *galS*: TF

adZ(u): *mglBAC*
- *mglBAC*: ABC-transporter (galactose)

nadZ: *galP*
- *galP*: MFS-transporter (galactose)

nadZ: *galETKM*
- *galETK*: enzymes for UDP-galactose biosynthesis
- *galM*: galactose-1-epimerase (enzyme that links lactose and galactose metabolisms)

Notes:
- GalS is homolog of the TF encoded in one of its two X-operons, *galR*. The additional X-operon is CRP

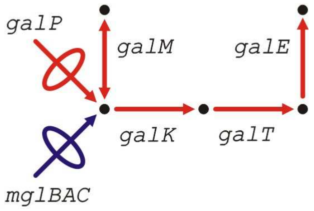

Y-op: *uxuR*
- *uxuR*: TF

adZ(u): *uxuAB*
- *uxuAB*: enzymes in fructuronate degradation pathway

adZ(2nd): *gntP*
- *gntP*: GNT-transporter (fructuronate/gluconate)

nadZ: *uidABC*
- *uidA*: enzyme in glucuronide degradation pathway
- *uidB*: GPH-transporter (glucuronide)
- *uidC*: membrane protein associated to *uidB*

Notes:
- UxuR is homologue of the TF encoded in one of its two X-operons, *exuR*. The additional X-operon is CRP
- *uxuAB* and *gntP* are divergent operons
- *uxaC* is regulated by ExuR. This gene is in the genome neighborhood of *exuR*

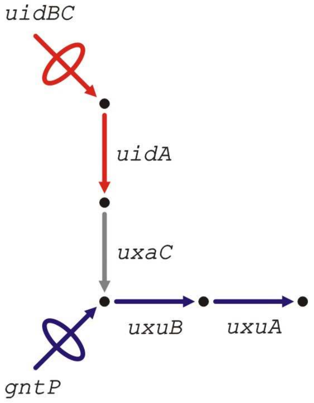

Y-op: *idnDOTR*
- *idnDO*: enzymes in idonate degradation pathway
- *indT*: GNT-transporter (idonate/gluconate)
- *idnR*: TF

adZ(d): *idnK*
- *idnK*: enzyme in idonate degradation pathway

nadZ: *gntKU*
- *gntK*: enzyme in idonate degradation pathway
- *gntU*: GNT-transp. (gluconate)

Notes:
- There are multiple homologies between the *idn* and *gnt* genes: *idnT* and *gntU* are homologs and so are *idnK* and *gntK*. Moreover, *idnR* is homolog of the TF encoded in one of its two X-operons, *gntR*, which is located upstream of *gntKU* in the genome. The additional X-operon is CRP

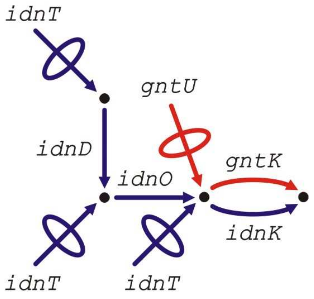

Y-op: *nagBACD*
- *nagBA*: enzymes in N-acetylglucosamine degradation pathway
- *nagC*: TF
- *nagD*: ribonucleotide monophosphatase

adZ(d): *nagE*
- *nagE* (+ *ptsHI*): PTS-transp. (N-acetylglucosamine)

nadZ: *manXYZ*
- *manXYZ* (+ *ptsHI*) : PTS-transporter (hexoses as N-acetylglucosamine)

nadZ: *chbBCARFG*
- *chbBCA* + (*ptsHI*): PTS-transporter (chitobiose)
- *chbR*: TF
- *chbF*: enzyme in chitobiose degradation pathway
- *chbG*: uncharacterized

nadZ: *nanC-yjhT*
- *nanC*: OmpG-channel(N-acetylneuraminic acid)
- *yjhT*: uncharacterized

Notes:
- *chbBCARFG* is an autoregulated operon
- see Notes for *dgsA* system

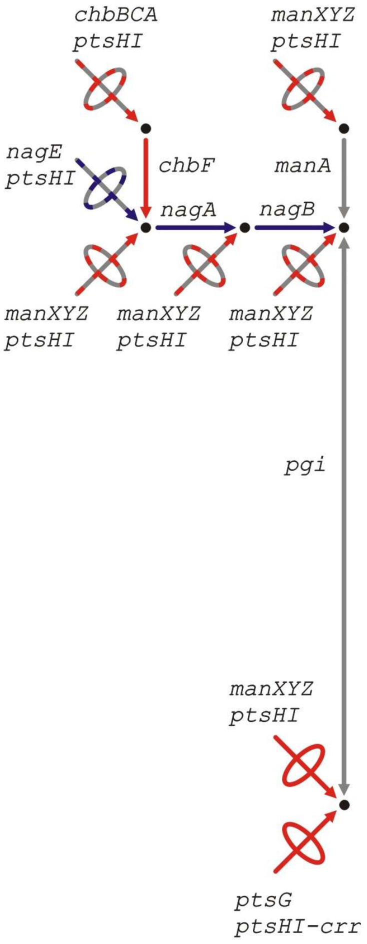

Y-op: *dgsA*
- *dgsA*: TF

nadZ: *malT*
- *malT*: TF

nadZ: *manXYZ*
- *manXYZ* (+ *ptsHI*) : PTS-transporter (hexoses as glucose)

nadZ: *ptsG*
- *ptsG* (+ *ptsHI-crr*): PTS-transporter (glucose)

nadZ: *ptsHI-crr*
- *ptsHI-crr*: PTS-transporter (non-specific-sugar components)

Notes:

- *dgsA* and *nagBACD* system (above) are very related: *nagC* and *dgsA* are homologs, and so are *nagE* and *ptsG*; pathways encoded in both systems are closely located in the metabolism and they use the same type of transporters (PTS)

Non-autoregulated Y-operon

Y-op: *glpEGR*
- *glpE*: thiosulfate sulfurtransferase
- *glpG*: intramembrane serine protease
- *glpR*: TF

adZ(d): *glpD*
- *glpD*: glycerol dehydrogenase (aerobic)

nadZ: *glpABC*
- *glpABC*: glycerol dehydrogenase (anaerobic)

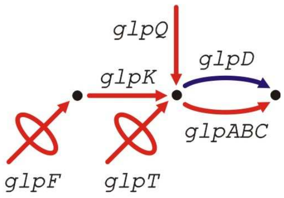

nadZ: *glpTQ*
- *glpT*: MFS-transporter (glycerol-3-P)
- *glpQ*: periplasmic transport associated enzyme

nadZ: *glpFKX*
- *glpF*: MIP-channel (glycerol)
- *glpK*: enzyme for glycerol degradation
- *glpX*: fructose 1,6-bisphosphatase (glycolisis enzyme)

Notes:
- *glpD* and *glpA* are homologs

Y-op: *dcuSR*
- *dcuSR*: 2-component system (anaerobic fumarate respiration)

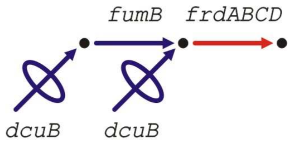

adZ(u): *dcuB-fumB*
- *dcuB*: DCU-transporter (dicarboxylates as fumarate)
- *fumB*: fumarase (anaerobic respiration)

nadZ: *frdABCD*
- *frdABCD*: fumarate reductase (anaerobic respiration)

Y-op: *cbl*
- *cbl*: TF

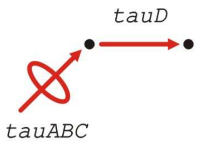

nadZ: *tauABCD*
- *tauABC*: ABC-transporter (taurine)
- *tauD*: taurine dehydrogenase

Notes:
- Cbl is homologue of the TF encoded in its only X-operon, *cysB*

Y-op: *malT*
- *malT*: TF

adZ(d): *malPQ*
- *malPQ*: enzymes for maltose and maltodextrins metabolism

nadZ: *malK-lamB-malM*
- *malK* (+ *malEFG*): ABC-transporter (maltose)
- *lamB*: sugar porin (maltose and maltodextrins)
- *malM*: periplasmic protein

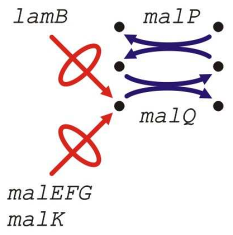

nadZ: *malEFG* (see *malK-lamB-malM*)

nadZ: *malS*
- *malS*: periplasmic maltohexaose transport associated enzyme

Notes:
- *malEFG* and *malK-lamB-malM* are divergent operons: the encoded ABC transporter and porin constitute the maltose/maltodextrin transport system

---

Y-op: *hupA*
- *hupA*: TF

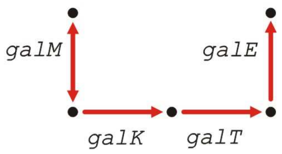

nadZ: *galETKM*
- *galETK*: enzymes for UDP-galactose biosynthesis
- *galM*: galactose-1-epimerase (enzyme that links lactose and galactose metabolisms)
