# Supplementary material for: What Determines the Assembly of Transcriptional Network Motifs in Escherichia coli?
Source: PLoS One. 2008 Nov 6;3(11):e3657. doi: 10.1371/journal.pone.0003657 (PMC2577066; doi:10.1371/journal.pone.0003657)
Supplement: Table S1 — Classification of the 230 FFLs in the network based on the connectivity of their respective X- and Y -TFs. LC, MC and HC for low-, medium- and highconnectivity classes, respectively. We also distinguished between autoregulated (curved arrow) and non-autoregulated (crossed-curved arrow) TFs, and those belonging to first (1st-L) and lower-layers (low-L). Small numbers denote number of instances in each subgroup (TFs only regulating their own operon are not considered; Y -elements belong to lower layers of the transcriptional network). The use of the “central unit” association implies an alternative classification of FFLs based on the number of nonadjacent regulated operons. Following this criterion, exuR, nagBACD and malT, all regulating one adjacent operon and four nonadjacent ones, are considered low connectivity operons. The minor differences introduced by this latter classification -which is the one used in Fig. 2.A, main text- are enclosed in parentheses. (0.01 MB PDF) [file pone.0003657.s002.pdf]

			***X*-TF**												
			LC				MC				HC				
			1st-L		low-L		1st-L		low-L		1st-L		low-L		
			$\circlearrowright$	$\not\circlearrowright$	$\circlearrowright$	$\not\circlearrowright$	$\circlearrowright$	$\not\circlearrowright$	$\circlearrowright$	$\not\circlearrowright$	$\circlearrowright$	$\not\circlearrowright$	$\circlearrowright$	$\not\circlearrowright$	
			15(16)	30	21(22)	13(14)	5(4)	5	7(6)	4(3)	7	1	9	6	total
***Y*-TF**	LC	$\circlearrowright$	0(1)	3	0	1	1(0)	2	1	1	27(31)	0	2	7	45(49)
		$\not\circlearrowright$	0	0	0	0	1	0	0	0	14(18)	0	1	3	19(23)
	MC	$\circlearrowright$	0	0	0	1	0	6	0	0	13(9)	0	1	8	29(25)
		$\not\circlearrowright$	0	0	0	0	0	0	0	0	9(5)	0	5	0	14(10)
	HC	$\circlearrowright$	1	0	0	0	0	0	1	0	29	0	20	10	61
		$\not\circlearrowright$	0	0	0	0	0	2	0	0	53	0	2	5	62
		total	1(2)	3	0	2	2(1)	10	2	1	145	0	31	33	230

Table S1
