# Supplementary material for: What Determines the Assembly of Transcriptional Network Motifs in Escherichia coli?
Source: PLoS One. 2008 Nov 6;3(11):e3657. doi: 10.1371/journal.pone.0003657 (PMC2577066; doi:10.1371/journal.pone.0003657)
Supplement: Table S2 — Relative orientation between upstream/downstream adjacent genes (→) and TRN operons (⇒). Upstream divergent orientation (←⇒) is particularly enriched. Curved arrow, operons encoding an autoregulated TF; crossed-curved arrow, operons encoding a non-autoregulated TF; low curved arrow, operons encoding an autoregulated low-connectivity TF; low crossed-curved arrow, operons encoding a non-autoregulated low-connectivity TF; not(low curved arrow), operons encoding a TF of the TRN excluding autoregulated low-connectivity ones. (0.01 MB PDF) [file pone.0003657.s003.pdf]

set	N	$\rightarrow\Rightarrow$	$\leftarrow\Rightarrow$	$\Rightarrow\rightarrow$	$\Rightarrow\leftarrow$	p
TRN	681	43.8	56.2	51.0	49.0	0.0013
$\circlearrowright$	76	**36.8**	**63.2**	51.3	48.7	0.02
$\not\circlearrowright$	59	47.5	52.5	45.8	54.2	0.41
low $\circlearrowright$	48	**29.2**	**70.8**	43.8	56.2	0.003
low $\not\circlearrowright$	43	48.8	51.2	46.5	53.5	0.51
not(low $\circlearrowright$)	87	48.3	51.7	51.7	48.3	0.43

Table S2
