# Supplementary material for: What Determines the Assembly of Transcriptional Network Motifs in Escherichia coli?
Source: PLoS One. 2008 Nov 6;3(11):e3657. doi: 10.1371/journal.pone.0003657 (PMC2577066; doi:10.1371/journal.pone.0003657)
Supplement: Table S3 — First-layer AOs. LC, MC and HC for low-, medium- and high-connectivity classes respectively. In LC without adjacent regulation we distinguish the cases of polycistronic and monocistronic AOs. † d, divergent; u, unidirectional. ‡ Regulated second neighbors included. Calculations based only on microarray data enclosed in brackets. Ψ In those cases with adjacent regulation, we showed number of promoters corresponding to the autoregulated and the adjacent operon, respectively. (0.01 MB PDF) [file pone.0003657.s004.pdf]

| set | | AO | Orientation of adj. regulated operon † | Number of nonadjacent regulated op. ‡ | Number of promoters in central unit Ψ |
|---|---|---|---|---|---|
| **LC** | **adjacent regulation** | *acrR* | d | 0 | 1/1 |
| | | *agaR* | d | 1 | 1/1 |
| | | *cusRS* | d | 0 | 1/1 |
| | | *cynR* | d | 0 | 1/1 |
| | | *evgAS* | d | 1 [1] | 2/1 |
| | | *gcvA* | d | 1 | 1/1 |
| | | *hcaR* | d | 0 | 1/1 |
| | | *ilvY* | d | 0 | 1/1 |
| | | *mngR* | d | 0 | 1/1 |
| | | *pspF* | d | 1 | 3/1 |
| | | *soxR* | d | 1 | 1/1 |
| | | *torR* | d | 1 [2] | 1/1 |
| | **poly.** | *ada-alkB* | - | 2 | 2 |
| | | *emrRAB* | - | 0 | 1 |
| | | *qseBC* | - | 0 [1] | 2 |
| | **mono.** | *lrhA* | - | 2 | 1 |
| | | *putA* | - | 0 | 1 |
| | | *trpR* | - | 4 | 1 |
| **MC** | | *cysB* | - | 6 [1] | 1 |
| | | *exuR* | u | 4 | 1/1 |
| | | *iscRSUA* | - | 6 | 1 |
| | | *tyrR* | - | 7 | 1 |
| | | *phoBR* | - | 9 [1] | 1 |
| **HC** | | *argR* | - | 10 | 2 |
| | | *cpxRA* | d | 20 | 1/1 |
| | | *crp* | d | 161 [13] | 1/1 |
| | | *fnr* | - | 85 [7] | 1 |
| | | *lexA-dinF* | - | 19 [1] | 1 |
| | | *lrp* | - | 22 [10] | 1 |
| | | *phoPQ* | - | 19 | 2 |

# Table S3
