# Supplementary material for: What Determines the Assembly of Transcriptional Network Motifs in Escherichia coli?
Source: PLoS One. 2008 Nov 6;3(11):e3657. doi: 10.1371/journal.pone.0003657 (PMC2577066; doi:10.1371/journal.pone.0003657)
Supplement: Table S4 — Lower-layers AOs of low-connectivity class. When there is not adjacent regulation we distinguish the cases of polycistronic and monocistronic AOs. † d, divergent; c, convergent; u, unidirectional. In the rhaSR case there is adjacent regulation over both the upstream and downstream neighbors. ‡ Regulated second neighbors included. Calculations based only on microarray data enclosed in brackets. Ψ In those cases with adjacent regulation, we showed number of promoters corresponding to the autoregulated and the adjacent operon, respectively. (0.01 MB PDF) [file pone.0003657.s005.pdf]

set	AO	Orientation of adj. regulated operon †	Number of nonadjacent regulated op. ‡	Number of promoters in central unit Ψ
**adjacent regulation**	*araC*	d	3	1/1
	betIBA	d	0	1/1
	fecIR	u	0	1/1
	galS	u	2	1/1
	glcC	d	0	1/1
	hypABCDE-fhlA	d	3	2/1
	idnDOTR	d	1	1/1
	malI	d	0	1/1
	melR	d	0	1/1
	metR	d	2 [1]	2/1
	prpR	d	0	1/1
	rhaSR	d,c	0	1/1
	uxuR	u	2	1/1
	xylFGHR	d	0	2/1
	zraSR	d	0	1/1
**poly.**	*chbBCARFG*	-	0	1
	gadAX	-	1 [9]	2
	hipBA	-	0	1
	hyfABCDEFGHIR-focB	-	0	1
	lctPRD (*lldPRD*)	-	0	2
	mdtABCD-baeSR	-	3	1
	mtlADR	-	0	1
	nikABCDER	-	0	2
	pdhR-aceEF-lpdA	-	2	3
	rbsDACBKR	-	0	1
	srlAEBD-gutM-srlR-gutQ	-	0	2
	tdcABCDEFG	-	0	1
**mono.**	*dgsA* (*mlc*)	-	4	2
	iclR	-	1	1
	nac	-	4 [2]	1

Table S4
