# Supplementary material for: What Determines the Assembly of Transcriptional Network Motifs in Escherichia coli?
Source: PLoS One. 2008 Nov 6;3(11):e3657. doi: 10.1371/journal.pone.0003657 (PMC2577066; doi:10.1371/journal.pone.0003657)
Supplement: Table S5 — Lower-layers AOs of medium- (MC) and high-connectivity (HC) classes. † d, divergent; u, unidirectional. ‡ Regulated second neighbors included. Calculations based only on microarray data enclosed in brackets. Ψ In those cases with adjacent regulation, we showed number of promoters corresponding to the autoregulated and the adjacent operon, respectively. ¶ cmk-rpsA-ihfB and thrS-infC-rpmI-rplT-pheMST-ihfA, encoding the two components of the transcription factor IHF, counted as a single node in the network (see the first section of text S1). (0.01 MB PDF) [file pone.0003657.s006.pdf]

set	AO	Orientation of adj. regulated operon †	Number of nonadjacent regulated op. ‡	Number of promoters in central unit Ψ
**MC**	*cytR*	-	8	1
	dnaAN-recF	-	5	8
	gadE	u	5 [8]	3/1
	glnALG	-	5 [7]	3
	nagBACD	d	4	3/1
	oxyR	-	8 [1]	1
	rcsA	-	6 [1]	1
**HC**	*dusB-fis*	-	54 [8]	1
	fldA-fur	-	31 [4]	4
	fliAZY	u	15	2/1
	hns	-	20 [21]	1
	marRAB	-	15 [1]	1
	purR	-	15 [2]	1
	rpoE-rseABC	-	51	3
	soxS	-	15 [1]	1
	cmk-rpsA-ihfB ¶<br>*thrS-infC-rpmI-rplT-pheMST-ihfA* ¶	-	56 [7]	4<br>7

Table S5
