# Supplementary material for: What Determines the Assembly of Transcriptional Network Motifs in Escherichia coli?
Source: PLoS One. 2008 Nov 6;3(11):e3657. doi: 10.1371/journal.pone.0003657 (PMC2577066; doi:10.1371/journal.pone.0003657)
Supplement: Table S6 — General features of SO and CP networks. Curved arrow, operons encoding an autoregulated TF (autoregulated operons); crossed-curved arrow, operons encoding a non-autoregulated TF. Operons encoding a TF that only regulates its own operon in parentheses. (0.01 MB PDF) [file pone.0003657.s007.pdf]

	SO	CP
nodes	423	681
non-autoregulatory interactions	519	1109
$\circlearrowright + \not\circlearrowright$	116	135
$\circlearrowright + \not\circlearrowright$ (first layer)	81	66
$\circlearrowright + \not\circlearrowright$ (lower layers)	35	69
$\circlearrowright$	59 (10)	76 (12)
$\circlearrowright$ (first layer)	35	30
$\circlearrowright$ (lower layers)	24	46
$\circlearrowright$ (% first-layer)	43.2	45.4
$\circlearrowright$ (% lower-layers)	68.6	66.7

Table S6
