# Supplementary material for: What Determines the Assembly of Transcriptional Network Motifs in Escherichia coli?
Source: PLoS One. 2008 Nov 6;3(11):e3657. doi: 10.1371/journal.pone.0003657 (PMC2577066; doi:10.1371/journal.pone.0003657)
Supplement: Table S7 — Comparison between autoregulated operons in SO and CP networks. An autoregulated operon in the CP network can be autoregulated (curved arrow), non-autoregulated (crossed-curved arrow) or absent (Abs) in the SO network, and conversely. We specified those operons located in first and lower network layers. Operons appearing in the network only as target operons in parentheses. (0.01 MB PDF) [file pone.0003657.s008.pdf]

SO	CP	cases	SO first lay.	SO lower lay.	CP first lay.	CP lower lay.
$\circlearrowright$	$\circlearrowright$	50	29	21	20	30
$\circlearrowright$	$\not\circlearrowright$	6	3	3	0	2+(4)
$\circlearrowright$	Abs	3	3	0	-	-
$\not\circlearrowright$	$\circlearrowright$	12	9	2+(1)	3	9
Abs	$\circlearrowright$	14	-	-	7	7

Table S7
