# Supplementary material for: What Determines the Assembly of Transcriptional Network Motifs in Escherichia coli?
Source: PLoS One. 2008 Nov 6;3(11):e3657. doi: 10.1371/journal.pone.0003657 (PMC2577066; doi:10.1371/journal.pone.0003657)
Supplement: Table S8 — Coherent and incoherent FFLs in SO and CP networks (as defined in ref. [2], text S1). Coh: coherent FFLs; Inc: incoherent FFLs, Other: FFLs with at least one dual-type interaction (see also note 3 in text S1). (0.00 MB PDF) [file pone.0003657.s009.pdf]

	SO	CP
Coh-1	28	66
Coh-2	2	15(+1 pseudo)
Coh-3	4	6
Coh-4	1	9
Inc-1	5	24
Inc-2	0	8
Inc-3	1	2
Inc-4	1	13 (+1 pseudo)
Other	0	87
total	42	230 (+2 pseudo)

Table S8
