# Supplementary material for: What Determines the Assembly of Transcriptional Network Motifs in Escherichia coli?
Source: PLoS One. 2008 Nov 6;3(11):e3657. doi: 10.1371/journal.pone.0003657 (PMC2577066; doi:10.1371/journal.pone.0003657)
Supplement: Table S9 — Distribution of operons per layer in SO and CP networks. We showed explicitely the distribution of autoregulated (curved arrow) and non-autoregulated TF (crossed-curved arrow). † The two components of the marRAB-rob loop are considered to be located both in the 6th layer. (0.01 MB PDF) [file pone.0003657.s010.pdf]

	**SO network**			**CP network**		
layer	operons	↻	∅	operons	↻	∅
1	81	35	46	66	30	33
2	233	17	8	177	20	10
3	87	5	3	113	4	3
4	10	2	0	88	6	1
5	12	0	0	65	7	3
6†				94	6	4
7				49	2	2
8				14	1	0
9				15	0	0

Table S9
