# Supplementary material for: What Determines the Assembly of Transcriptional Network Motifs in Escherichia coli?
Source: PLoS One. 2008 Nov 6;3(11):e3657. doi: 10.1371/journal.pone.0003657 (PMC2577066; doi:10.1371/journal.pone.0003657)
Supplement: Table S10 — Characterization of low-connectivity Y -TFs establishing FFLs with at least one nadZ. First and second columns: Y and X TFs -homolog pairs in bold (two-component systems are also shown). Third and fourth columns: functional characterization of proteins in the central unit and corresponding nadZs labeled with numbers. This also shows the homology relationship -highlighted by same color- between genes in nadZs and those in the associated central unit. Abbreviations: TF, transcriptional factor; 2c, two-component system; E, Enzyme; T, transporter; PTAE, periplasmic transportassociated enzyme; U, uncharacterized protein; NP, near pathway, products acting in regions of the metabolic pathway near those of the central unit; RP: redundant pathway, including proteins which constitute multienzymatic complexes with those encoded in the central unit; P: pathway, sometimes there is no pathway encoded in the central unit, but in the nadZs. See Appendix in text S1 for further details. (0.00 MB PDF) [file pone.0003657.s011.pdf]

Y-TF	X-TFs	central-unit products	nonadjacent Z-operons products
AraC	CRP	TF, E	1: T; 2: T; 3: T
**Cbl**	**CysB**	TF	1: P[E, T]
DcuS-DcuR (2c)	Fnr, NarL	2c, E, T	1: NP[E]
DgsA	CRP	TF	1: TF; 2: T; 3: T; 4: T
**GadX**	CRP, **GadW**, GadE, RpoS	TF, E	1: RP[E, T]
**GalS**	CRP, **GalR**	TF, T	1: T; 2: P[E]
GlpR	CRP	TF, E, E	1: RP[E]; 2: T, PTAE; 3: NP[T, E], E
HU	CRP	TF	1: P[E]
FhlA	Fnr, IHF, RpoN	TF, E, E	1: RP[TF, E, T]; 2: RP[E]; 3: RP[E, E]
**IdnR**	CRP, **GntR**	TF, E, E, T	1: RP[E, T]
MalT	CRP	TF, E	1,2: T, T, U; 3: PTAE
**BaeS-BaeR** (2c)	**CpxA-CpxR** (2c)	2c, T, T	1: T
NagC	CRP	TF, E, T	1: T; 2: NP[TF, E, T]; 3: T
PdhR	CRP, Fnr, ArcA (pseudo FFL)	TF, E	1: NP[TF, E, T]; 2: NP[E]
**UxuR**	CRP, **ExuR**	TF, E, T	1: NP[E, T]

Table S10
