# Supplementary figures and images for: What Determines the Assembly of Transcriptional Network Motifs in Escherichia coli?
Source: PLoS One. 2008 Nov 6;3(11):e3657. doi: 10.1371/journal.pone.0003657 (PMC2577066; doi:10.1371/journal.pone.0003657)

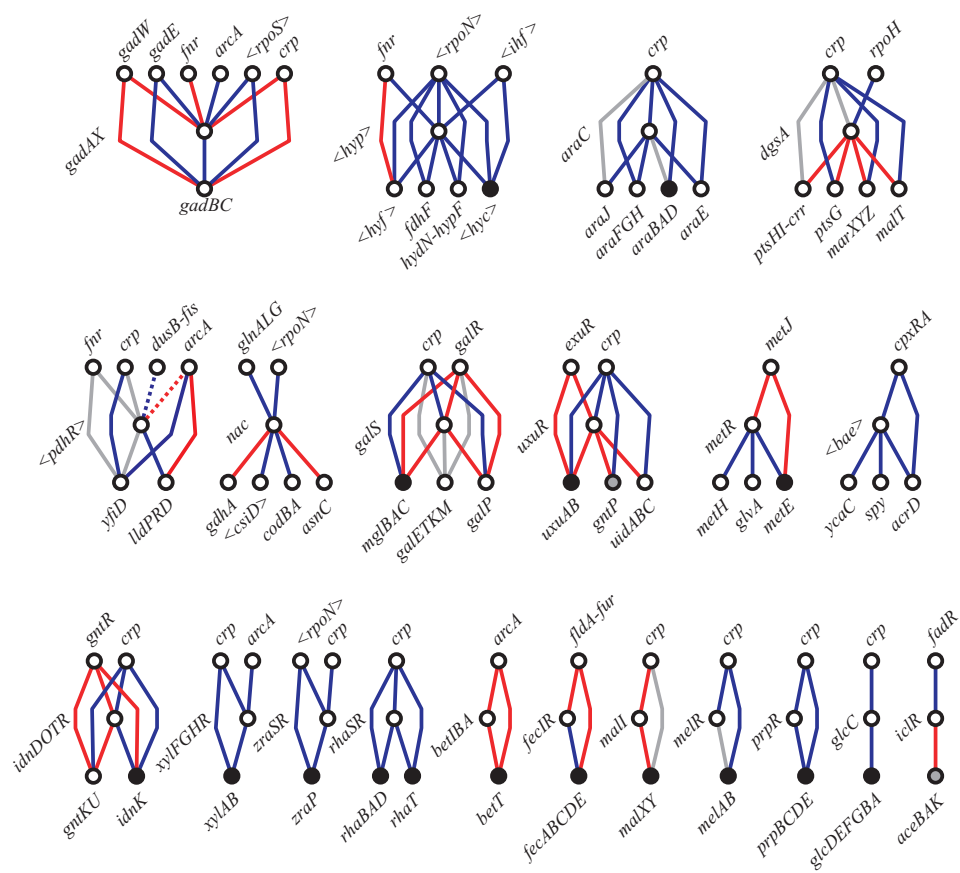

Figure S1

Supplement: Figure S1 — Regulatory links associated to lower-layers operons encoding a low-connectivity autoregulated TF (1≤out-degree<5). We showed incoming and outgoing regulations and also those additional ones to describe FFLs (X-Z interactions). Edges color code: blue, activation; red, repression; gray, dual regulation. Z-operons filling color code: black, Z- and Y -operon are adjacent; gray, Z and Y are second neighbors; white, Z and Y are not adjacent. Dashed lines denote links where the TF encoded in the autoregulated operon is not affected by the regulation. This particularly applies to the regulation of pdhR-aceEFlpdA by arcA, and leads to the constitution of two pseudo-FFLs. Abbreviations: , nlpD-rpoS; , hypABCDE-fhlA; , hycABCDEFGHI; , hyfABCDEFGHIJR-focB; , lptB-rpoN-yhbH-ptsN-yhbJ-npr; , cmkrpsA-ihfB; , csiD-ygaF-gabDTP; , mdtABCD-baeSR; , pdhRaceEF- lpdA; , srlAEBD-gutM-srlR-gutQ; , tdcABCDEFG. Averaged FFLness: = 0.77. (0.02 MB PDF) [file pone.0003657.s012.pdf]

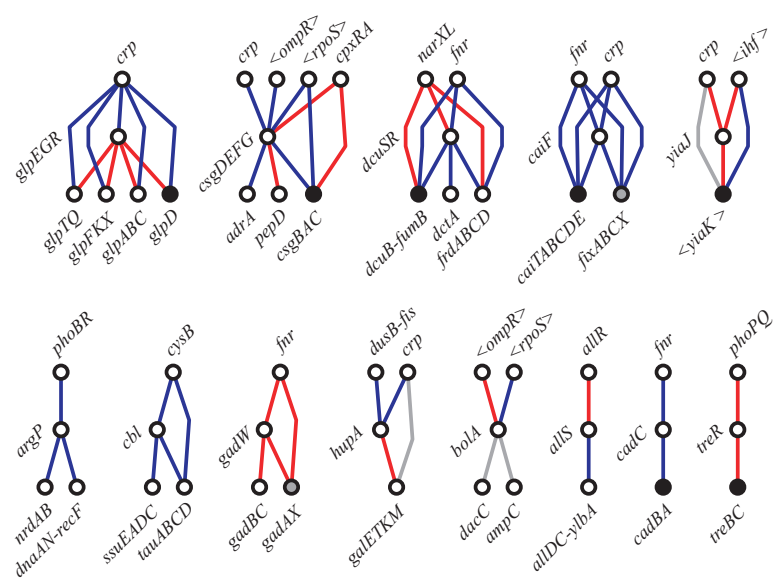

Figure S2

Supplement: Figure S2 — Regulatory links associated to lower-layers operons encoding a low connectivity non-autoregulated TF (out-degree<5). Abbreviations: , ompR-envZ; , yiaKLMNO-lyxK-sgbHUE, rest of abbreviations as before. Color coding as in Figure S1. = 0.46. (0.02 MB PDF) [file pone.0003657.s013.pdf]

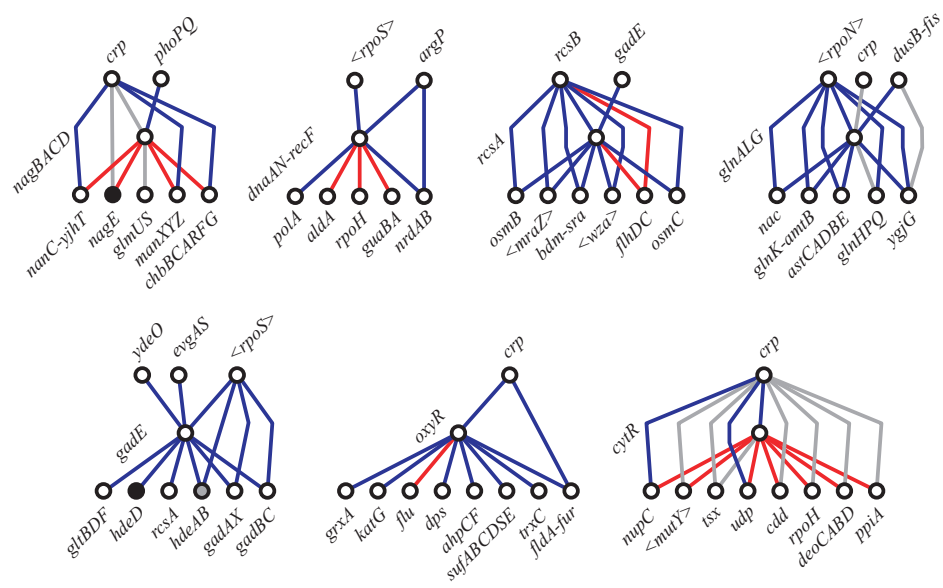

Figure S3

Supplement: Figure S3 — Regulatory links associated to lower-layers operons encoding a medium connectivity autoregulated TF (5≤out-degree<10). In the alternative classification of TFs based on the number of nonadjacent regulated operons nagBACD is considered a low-connectivity operon. Maximal FFLness of rcsA, glnALG and cytR corresponds to pairs (X,Y) in which the action of one TF totally relies on the presence of its partner (RcsA on RcsB, RpoN on NtrC -encoded in glnG- and CytR on CRP). Abbreviations: , mraZW-ftsLI-murEF-mraYmurD- ftsW-murGC-ddlB-ftsQAZ; , wza-wzb-wzc-wcaAB; , mutYyggX- mltC-nupG, rest of abbreviations as before. Color coding as in Figure S1. = 0.66. (0.02 MB PDF) [file pone.0003657.s014.pdf]

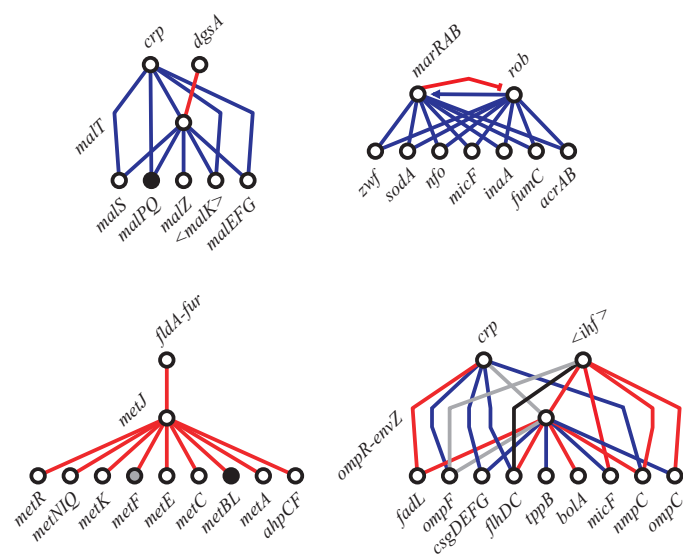

Figure S4

Supplement: Figure S4 — Regulatory links associated to lower-layers operons encoding a medium connectivity non-autoregulated TF (5≤out-degree<10). In the alternative classification of TFs based on the number of nonadjacent regulated operons malT is considered a low-connectivity operon. The type of transcriptional interaction between cmk-rpsA-ihfB and flhDC is not known (in black). Abbreviations: , malK-lamB-malM, rest of abbreviations as before. Color coding as in Figure S1. = 0.39. (0.01 MB PDF) [file pone.0003657.s015.pdf]
